# Supplementary material for: Activity of Gut-Derived Nisin-like Lantibiotics against Human Gut Pathogens and Commensals
Source: ACS Chem Biol. 2024 Jan 31;19(2):357–69. doi: 10.1021/acschembio.3c00577 (PMC10877564; doi:10.1021/acschembio.3c00577)
Supplement: Supplementary file 1 — cb3c00577_si_001.pdf [file cb3c00577_si_001.pdf]

Supporting Information for Activity of Gut-derived Nisin-like Lantibiotics Against Human Gut Pathogens and Commensals

Zhenrun J. Zhang,[‡*1,2] Chunyu Wu,[‡3] Ryan Moreira,[4] Darian Dorantes,[3] Téa Pappas,[1] Anitha Sundararajan,[1] Huaiying Lin,[1] Eric G. Pamer,[*1,5] Wilfred A. van der Donk*[3,4]

1 Duchossois Family Institute, University of Chicago, Chicago, IL 60637, USA
2 Department of Microbiology, University of Chicago, Chicago, IL 60637, USA
3 Department of Biochemistry, University of Illinois at Urbana–Champaign, Urbana, IL 61801, USA
4 Department of Chemistry, The Howard Hughes Medical Institute, University of Illinois at Urbana–Champaign, Urbana, IL 61801, USA
5 Departments of Medicine and Pathology, University of Chicago, Chicago, IL 60637, USA
‡ These authors contributed equally to this study.
* Corresponding authors: Zhenrun J. Zhang (zjzhang@bsd.uchicago.edu); Eric. G. Pamer (egpamer@bsd.uchicago.edu); Wilfred A. van der Donk (vddonk@illinois.edu)

Methods

General methods

Chemical reagents and media components used in this study were purchased from Sigma-Aldrich or Thermo Fisher Scientific, unless otherwise specified. Oligonucleotides and enzymes were purchased from Integrated DNA Technologies (IDT) and New England Biolabs (NEB), respectively. Polymerase chain reaction (PCR) amplifications were carried out using Q5 polymerase (NEB) on an automated thermocycler (C1000, Bio-Rad). DNA sequencing was performed using appropriate primers by ACGT, Inc. MALDI-TOF MS analyses were conducted at the Mass Spectrometry Facility at UIUC using a Bruker UltrafleXtreme MALDI TOF/TOF spectrometer (Bruker Daltonics). For MALDI−TOF MS analysis, samples were desalted using ZipTipC18 (Millipore) and spotted onto a MALDI target plate with a matrix solution usually consisting of a saturated aqueous solution of super DHB (2,5-dihydroxy benzoic acid; Sigma-Aldrich). Peptide purification was carried out by reversed-phase high performance liquid chromatography (RP−HPLC) on an Agilent 1260 Infinity II instrument equipped with a Macherey-Nagel C18 reverse-phase column (4.6 mm i.d. × 250 mm L). For RP−HPLC, solvent A was 0.1% TFA in H_2O, and solvent B was acetonitrile containing 0.1% trifluoroacetic acid (TFA). An elution gradient from 0% solvent B to 100% solvent B over 30 min was used unless specified otherwise.

Bioinformatic mining for gut-derived lantibiotics

Bioinformatic mining for gut-derived class I lantibiotics was performed similarly to methods previously reported.[1] The non-redundant protein records for bacteria in the NCBI RefSeq collection (February 2020) were searched with the LanC-Like Pfam HMM (PF05147.12) using HMMER3 with default settings. Proteins encoded within seven ORFs upstream and downstream

of the LanC-Like proteins were searched for precursor peptides using RODEO.[2] These precursor peptides were aligned against both nisin A and blauticin by BLAST, and candidates with less than 30% identity were filtered out. Multiple sequence alignments were performed on nisin A, blauticin and remaining candidate peptides, and close homologs were manually selected by using the following criteria: (a) the peptide length is less than or equal to 72; (b) they contain 5 ring-forming cysteine residues at similar relative locations to nisin A and blauticin. Sequences with different leader peptides but same core peptides were further consolidated.

Plasmid construction
The plasmids constructed for this study and their combined application for peptide production and purification are shown in Figure S2. The *Blautia producta SCSK* (BP_{SCSK}) $tRNA^{Glu}$ sequences were identified using the algorithm tRNAscan-SE.[3-4] For blauticin plasmid construction, pDuet1-His-BpcA and pCDFDuet1-BpcBC were synthesized by SynbioTechnologies. The genes BP_{SCSK} $tRNA^{Glu}$ and gluRS were constructed using synthetic gene fragment gBlocks (IDT), and amplified using primers for both inserts and backbones (Table S2). For blauticin analog or SAR mutant precursor peptide plasmid construction, the genes for precursors were constructed using synthetic gene fragment gBlocks (IDT), and amplified using primers for both inserts and backbones (Table S2).

Heterologous expression of peptides with pEVOL plasmids
Chemically competent *E. coli* BL21 (DE3) cells were transformed with combinations of the plasmids prepared (Figure S2) to co-express enzymes. Colonies were selected on LB agar plates containing the appropriate antibiotics listed for each plasmid. Single colonies were used to inoculate 5 mL of overnight starter cultures in LB with the appropriate antibiotic. The starter cultures were then used to inoculate 1 L of TB cultures containing the appropriate antibiotic, which were incubated at 37 °C with shaking (180 rpm) until they reached the beginning of the exponential growth phase (an OD_{600} of approximately 0.4). Cultures were then cooled at 4 °C for 15 min prior to the addition of L-arabinose to a final concentration of 0.1%. Cultures were placed into a 20 °C incubator and shaken (180 rpm) for at least 2 h until the culture reached the end of the exponential growth phase (an OD_{600} of approximately 1.2). The "slow growing time" that was maintained for a minimal of two hours before adding the next inducer was critical for the successful expression. Next, isopropyl β-D-1-thiogalactopyranoside (IPTG) was added to the culture to a final concentration of 0.5 mM. Cultures were placed into an 18 °C incubator and shaken overnight (180 rpm). Cells were harvested by centrifugation at 4,500 × g for 20 min. The culture media was decanted, and the cell pellet stored at −80 °C prior to purification.

Peptide purification
Cell pellet was suspended in 30 mL of lysis buffer (20 mM NaH_2PO_4, 500 mM NaCl, 0.5 mM imidazole, 20% glycerol, pH 7.5 at 25 °C) supplemented with Pierce Protease Inhibitor tablets (1 tablet per 1 L culture; Fisher Scientific) and benzonase (1 U for every 20 mL of lysate). The cell pellet was lysed by sonication (50% amplitude, 2 s pulse, 5 s pause, 15 min). Crude lysate was centrifuged at 24,000 ×g for 60 min at 4 °C and the supernatant was obtained. The supernatant was loaded to onto a His60 Ni superflow resin (Takara) equilibrated with lysis buffer, and cell lysate was applied three times to it under the action of gravity. After loading the sample, the resin was washed with two column volumes (CV) of washing buffer (4 M guanidine hydrochloride, 20 mM NaH_2PO_4, 300 mM NaCl, 30 mM imidazole, pH 7.5 at 25 °C), and eluted using 1 CV of LanA

Elution Buffer (4 M guanidine hydrochloride, 20 mM Tris HCl, pH 7.5 at 25 °C, 100 mM NaCl, 1 M imidazole). Peptides eluted from the Ni resin were concentrated and desalted using 3 kDa Amicon filters (Millipore). For leader peptide removal, the peptide (1:20 trypsin to peptide) was incubated with the trypsin protease in 50 mM Tris-HCl, pH 8 overnight at 37 °C. Lastly, the core peptides were further purified with HPLC using an Agilent 1200 instrument (Agilent) as specified in general methods. About 1.5 mg recombinant peptide was obtained after HPLC purification per L of *E. coli* culture.

Purification of blauticin from *Blautia producta* SCSK

Stationary anaerobic culture of BP_{SCSK} was inoculated 1:100 to 1 L BHIS. Culture was incubated on a heating plate at 37 °C with 220 rpm stirring for 2 days in an anaerobic chamber. Bacteria were pelleted with 15,000 x g centrifugation for 15 min. The supernatant was acidified with acetic acid to pH 5.5 and filtered through a 0.22 um filter. Filtered supernatant was loaded onto a HiTrap SP HP column using an AKTA Pure FPLC, which was eluted sequentially with 3 CV of distilled water, 200 mM NaCl, 400 mM NaCl, 600 mM NaCl, 800 mM NaCl, and 1 M NaCl. The 800 mM NaCl eluate was concentrated with a 3 kDa Amicon filter, followed by dialyzation with DPBS using a 3 kDa Amicon filter. Purification of blauticin was confirmed by SDS-PAGE, VRE inhibition in vitro assay, and MALDI-TOF MS.

Isolation of human gut commensal bacteria

Isolation and growth of commensal bacteria was done as previously reported.[5] Briefly, fresh donor fecal samples were transferred into an anaerobic chamber (Coy Labs) within 1 h of collection. The anaerobic chamber was maintained using a gas mix consisting of 5% hydrogen, 5% carbon dioxide and 90% nitrogen, and hydrogen was maintained at ~3.0% using an anaerobic gas infuser. Fecal samples were resuspended in pre-reduced PBS and plated on Columbia blood agar or brain-heart infusion (BHI – Difco, BD) agar plates in three serial 10-fold dilutions and incubated at 37 ^{o}C for 48 – 96 h. Isolated colonies were re-streaked for purity onto Columbia blood agar and frozen in pre-reduced 10% glycerol in PBS.

Bacterial growth

For broth cultures, isolates were grown in pre-reduced BHI supplemented with 5 g/L yeast extract (Difco, BD) and 0.1% L-cysteine under anaerobic condition.

Minimal Inhibition Concentration (MIC) assay

All MIC assays were performed under anaerobic condition. Stationary bacterial culture in BHIS (BHI supplemented with 5 g/L yeast extract and 0.1% L-cysteine) were inoculated 1:100 to wells containing 200 μL BHIS in U-bottom 96-well plates. Lantibiotics were dissolved in DPBS, and concentrations were determined by BCA assay (Pierce) according to the manufacturer’s protocol. Lantibiotic stock solutions were added to wells to reach desired final concentration, with 3-fold serial dilution across rows to create concentration gradients. Bacteria-inoculated wells without lantibiotics served as control. Plates were incubated at 37 ^{o}C for 48 h before being taken out of the anaerobic chamber for centrifugation. Plates were imaged with an iBright 1500 imager (Invitrogen). The lowest concentration of lantibiotics that caused no or significantly less bacterial pellets than those without lantibiotics were identified as MIC.

Liposome Permeabilization Assay

To prepare pyranine encapsulated liposomes, DOPC (25 mg/mL in $CHCl_3$, purchased from Avanti) was deposited into a vial and mixed with lipid II (dissolved in 2:1 $CHCl_3$: MeOH, prepared chemoenzymatically).[6] The solvent was removed using a stream of N_2 leaving a thin film. Residual solvent was removed by storing it overnight in a vacuum desiccator. The film was rehydrated in buffer (5 mM Tricine, 5 mM MES, 5 mM NaCl, 1 mM pyranine pH = 6.0) and suspended with vortexing. The suspension was subjected to freeze-thaw three times before extrusion through a 200 µm polycarbonate filter (21 passes). Excess pyranine was removed by passing the liposome solution through a Sephadex G-50 column previously equilibrated with buffer (5 mM Tricine, 5 mM MES, 5 mM NaCl, pH = 6.0). The eluted liposome solution containing the highest concentration of encapsulated pyranine (as determined visually with a UV lamp) was immediately placed on ice. Phospholipid concentration was determined by NMR as described previously.[7]

To measure liposome permeabilization, to a 96 well black polystyrene plate was added buffer (5 mM Tricine, 5 mM MES, 5 mM NaCl, pH = 8.0; 97 µL) and liposome solution (3 µL; 50 µM phospholipid concentration during assay). The wells were allowed to equilibrate for 8 min at room temperature then antibiotic was added. The antibiotic concentration in each well was 375 nM. Pyranine fluorescence was monitored for 30 min then Triton X-100 was added to a final concentration of 0.1% and pyranine fluorescence was monitored for an additional 5 min. The pyranine fluorescence signal was max-min normalized.

Lantibiotic resistance gene mining

Genomes of pathogens and human gut commensals investigated were retrieved from RefSeq (Table S3). Genomes were annotated using Prokka,[8] which is based on both Hidden Markov Model (HMM) search and BLAST search. Genes with consistent annotations were numerated in each genome. For lantibiotic resistance genes without consistent annotations, an HMM search with corresponding HMM profiles (Table S3) were performed with hmmsearch in HMMER 3.3.[9] In case of *mprF* and *nsr* genes where HMM profiles were not available, individual protein sequences were retrieved from Uniprot (Table S3) and were searched against genomes with phmmer in HMM 3.3.[9] *E*-value cutoffs were set at e^{-15} for both hmmsearch and phmmer.

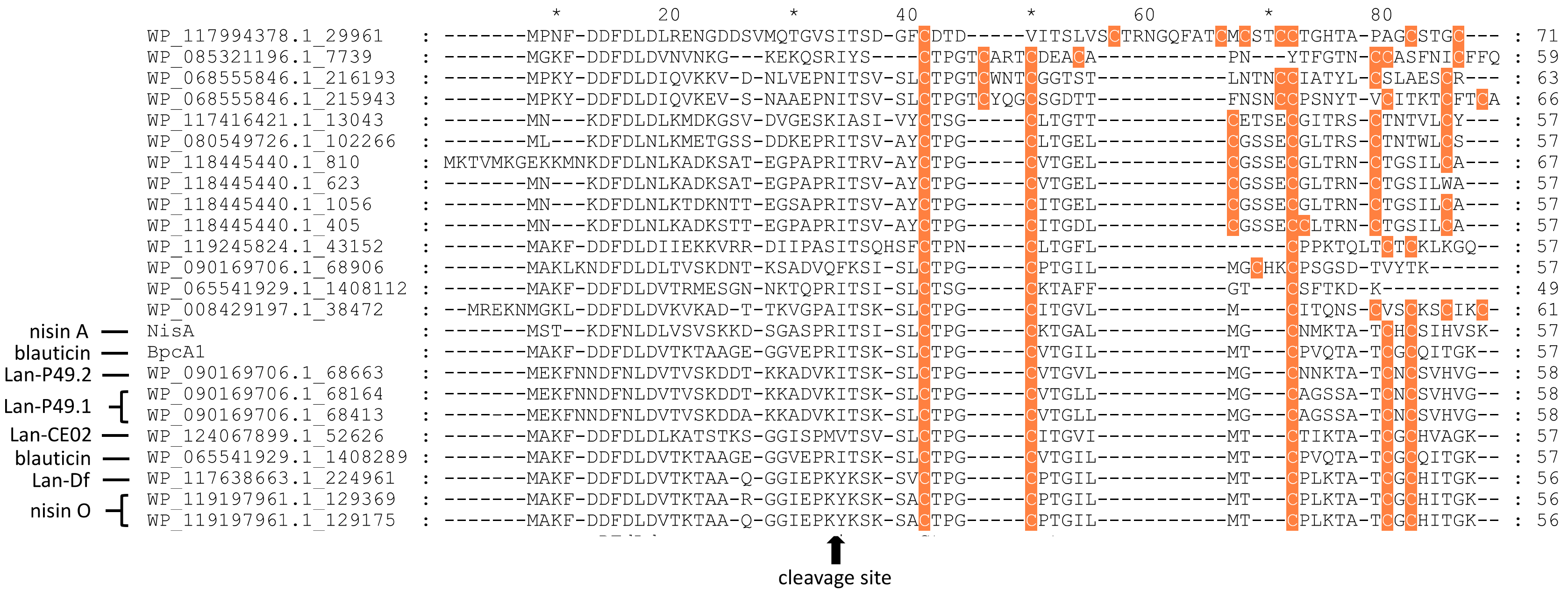

Fig. S1 Multiple Sequence Alignment (MSA) of nisin-like lantibiotic candidates discovered in the RefSeq database after length filtering. Cysteine residues that are important for thioether ring formation are colored in orange. The precursor peptides for nisin A (NisA) and blauticin (BpcA1) were included for comparison of cysteine positions as benchmarks for manual curation. The sequence length of each peptide is shown on the far right. Predicted protease cleavage sites of precursor peptides of nisin A and blauticin are marked at the bottom. Final nisin-like lantibiotic candidates are denoted on the left.

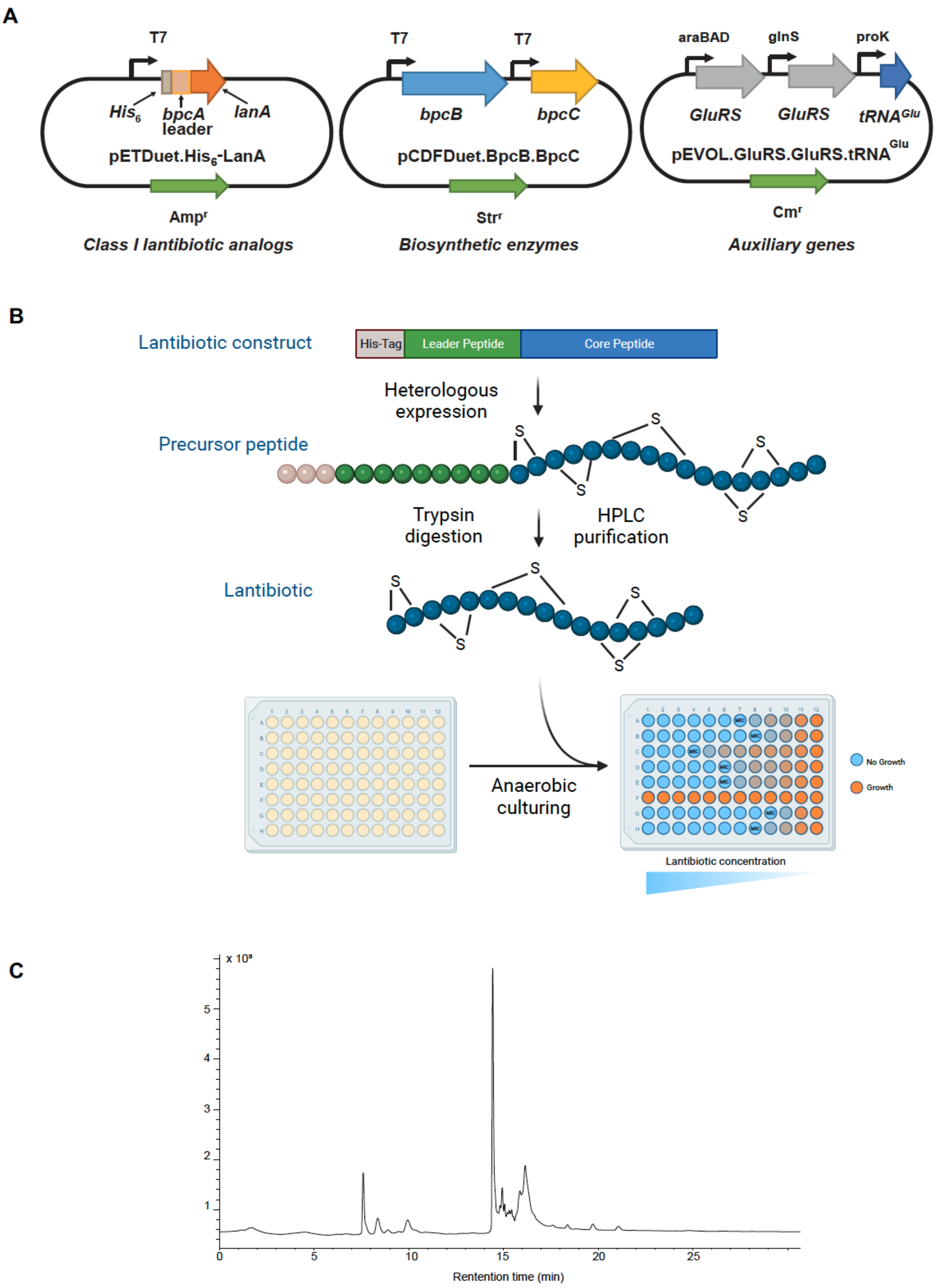
A
T7
His6
bpcA leader
lanA
pETDuet.His6-LanA
Ampr
Class I lantibiotic analogs
T7
T7
bpcB
bpcC
pCDFDuet.BpcB.BpcC
Strr
Biosynthetic enzymes
araBAD
glnS
proK
GluRS
GluRS
tRNAGlu
pEVOL.GluRS.GluRS.tRNAGlu
Cmr
Auxiliary genes
B
Lantibiotic construct
His-Tag
Leader Peptide
Core Peptide
Heterologous expression
Precursor peptide
Trypsin digestion
HPLC purification
Lantibiotic
Anaerobic culturing
No Growth
Growth
Lantibiotic concentration
C
x 10³
Rentention time (min)

D

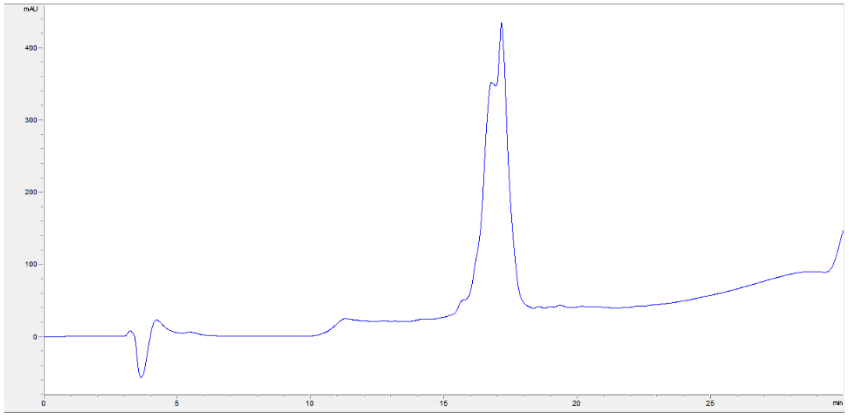

Fig. S2 Scheme of improved expression platform and lantibiotic production workflow. (A) Illustration of the three plasmids used in the expression platform. **(B)** Workflow of expression, purification, and MIC testing of each lantibiotic variant. Figure was created with biorender.com. **(C)** Representative HPLC trace of recombinant lanthipeptide following leader peptide removal but before HPLC purification (the blauticin trace is shown). **(D)** Representative HPLC trace (220 nm) of lanthipeptide after HPLC purification following leader peptide removal (Lan-Df as a mixture of dehydration states is shown).

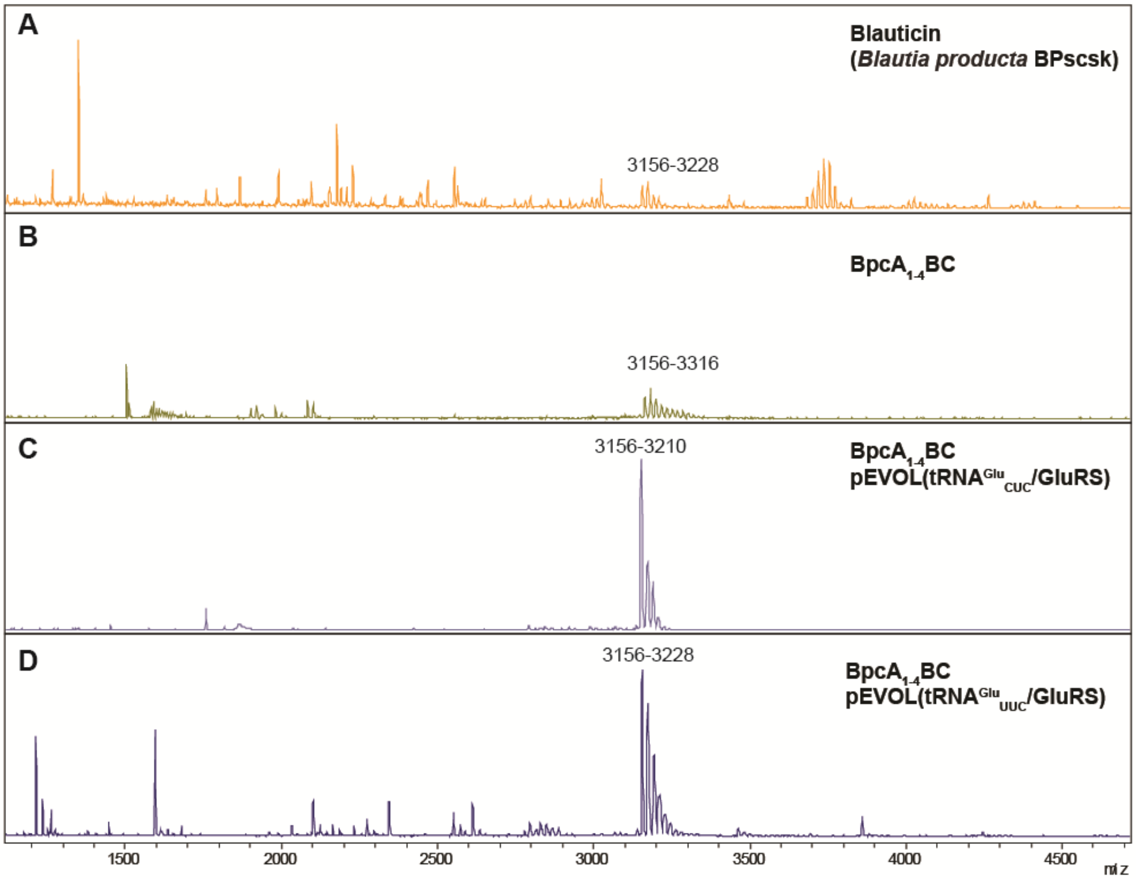

Fig. S3 MALDI-TOF MS analysis of heterologous production of blauticin using the pEVOL vector in *E. coli*. (A) MALDI-TOF MS analysis of blauticin isolated from BP_{SCSK}. (B-D) His_6-$BpcA_1$ isolated from the co-expression with BpcBC without (B) and with the pEVOL platform using $tRNA^{Glu}{}_{CUC}$ (C) or $tRNA^{Glu}{}_{UUC}$ (D) in *E. coli* after leader peptide removal by trypsin. 5-fold dehydrated $BpcA_1$ ($[M + H]^+$ m/z 3,228, calc. m/z 3,226); 6-fold dehydrated $BpcA_1$ ($[M + H]^+$ m/z 3,210, calc. m/z 3,208); 7-fold dehydrated $BpcA_1$ ($[M + H]^+$ m/z 3,192, calc. m/z 3,190); 8-fold dehydrated $BpcA_1$ ($[M + H]^+$ m/z 3,175, calc. m/z 3,172); 9-fold dehydrated $BpcA_1$ ($[M + H]^+$ m/z 3,156, calc. m/z 3,154). Peaks without labels are impurities from the producing organism.

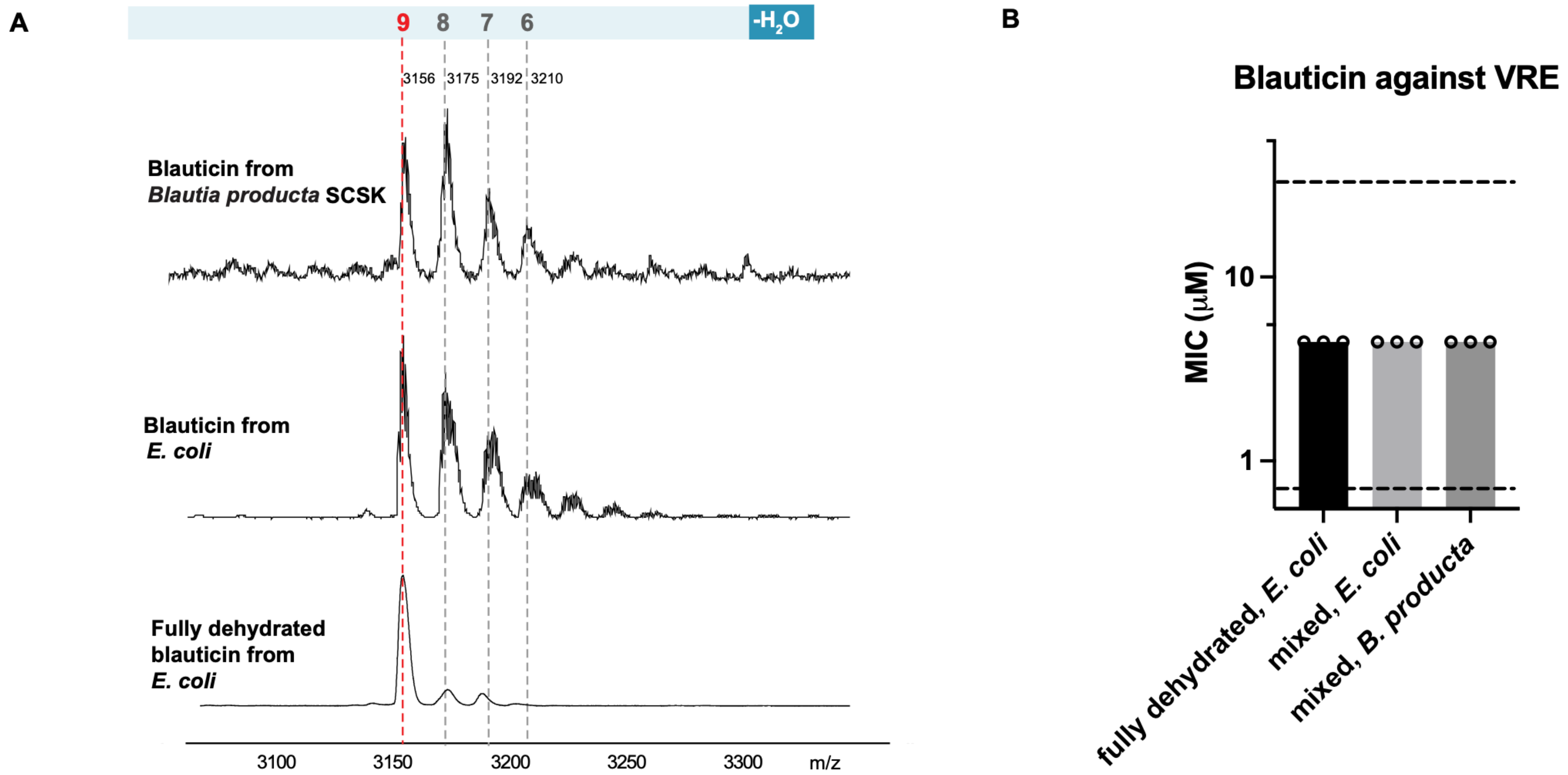

Fig. S4. Bioactivities of blauticin heterologously produced in *E. coli* and purified from its native host *B. producta*. (A) MALDI-TOF MS analysis of blauticin produced in *B. producta* SCSK, *E. coli* and fully dehydrated blauticin purified from *E. coli*. The numbers on top of the peaks indicate dehydration states for each peptide. Red numbers indicate full dehydration of all Ser/Thr residues. Observed masses for each peak are labeled on top. **(B)** MIC assay of blauticin in fully dehydrated form or mixed forms purified from *E. coli*, and blauticin in mixed forms from *B. producta* against Vancomycin-resistant *Enterococcus faecium* ATCC700221 (VRE). Each dot indicates one measurement of MIC value, determined by the lowest concentration that caused no or significantly less bacterial growth. Each bar indicates median MIC value. Upper and lower dashed lines indicate upper and lower limit of concentration tested, respectively.

A

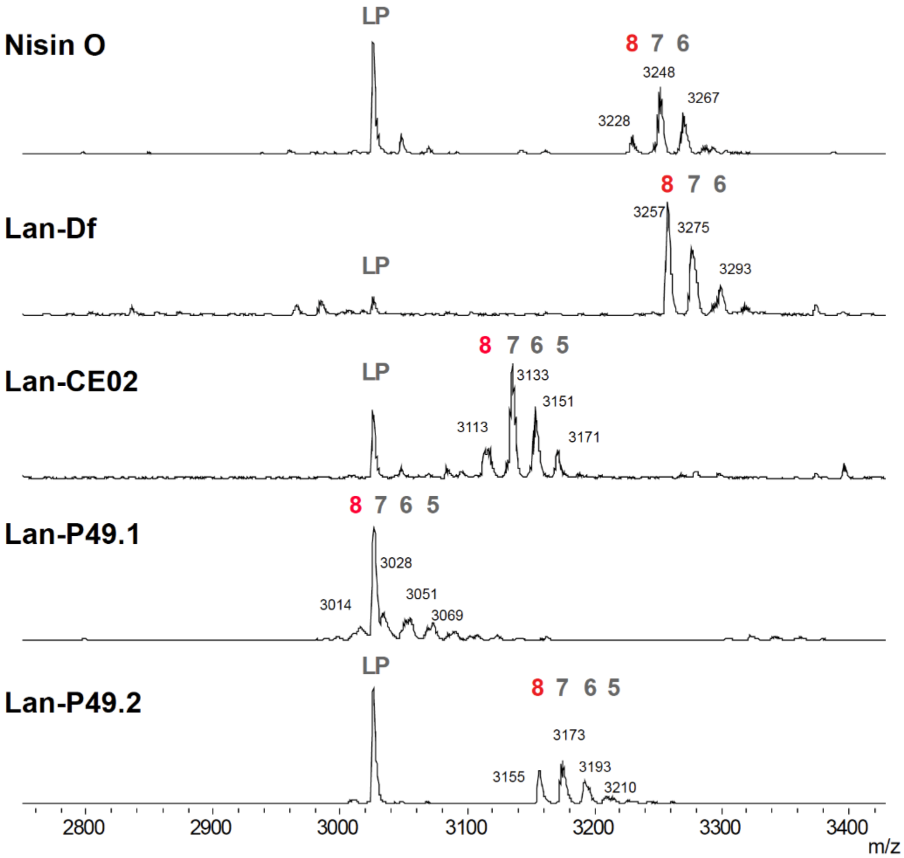

Nisin O
LP
8 7 6
3248
3267
3228
Lan-Df
8 7 6
3257
3275
3293
LP
Lan-CE02
8 7 6 5
LP
3133
3151
3113
3171
Lan-P49.1
8 7 6 5
3028
3014
3051
3069
Lan-P49.2
LP
8 7 6 5
3173
3193
3155
3210
2800
2900
3000
3100
3200
3300
3400
m/z

B

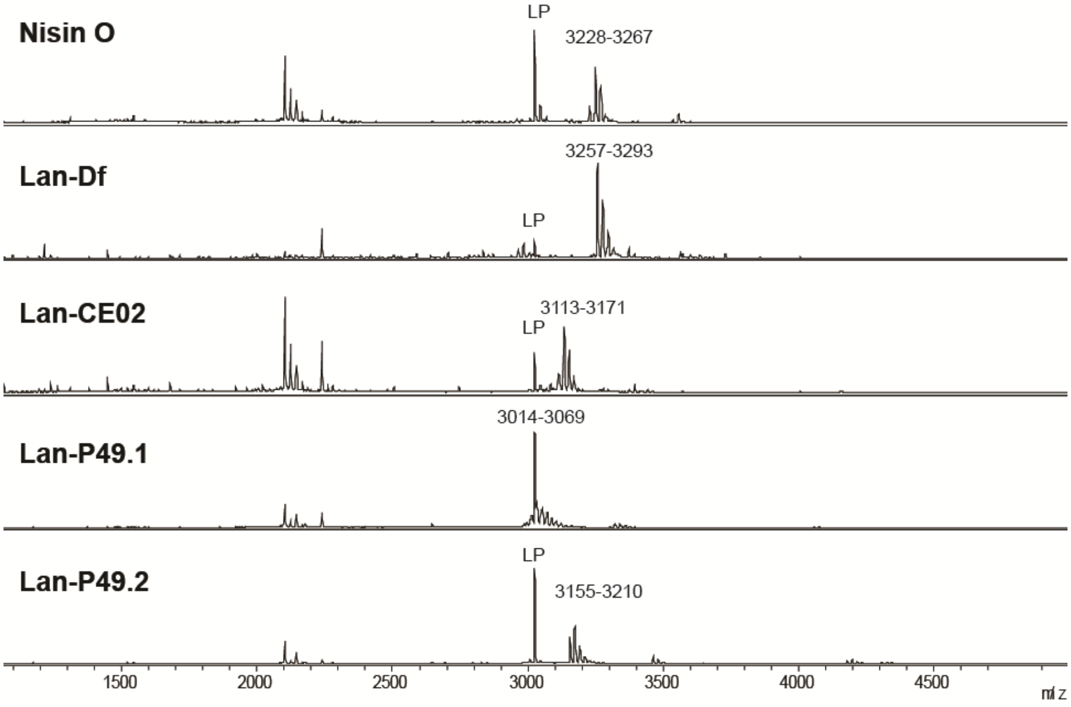

Nisin O
LP
3228-3267
Lan-Df
3257-3293
LP
Lan-CE02
3113-3171
LP
Lan-P49.1
3014-3069
Lan-P49.2
LP
3155-3210
1500
2000
2500
3000
3500
4000
4500
m/z

Fig. S5. MALDI-TOF MS analysis of nisin O, Lan-Df, Lan-CE02, Lan-P49.1, and Lan-P49.2 produced in *E. coli* after leader peptide cleavage but before HPLC purification. (A) The numbers on top of the peaks indicate dehydration states for each peptide. Red numbers indicate full dehydration of all Ser/Thr residues. Observed masses for each peak are labeled on top. LP, leader peptide. For Lan-P49.1, the peaks of the seven-fold dehydrated peptide and the leader peptide overlap. Observed and expected masses for each compound are listed in Table S1. Because of the limited resolution, some of the peaks are off by 1 Da from the calculated values, but the high resolution ESI spectra in Fig. S6 and the corresponding errors of the observed compared to the calculated values clearly establish the identify of the peptides. (**B**) Same compounds but with the window of the mass spectra widened.

A

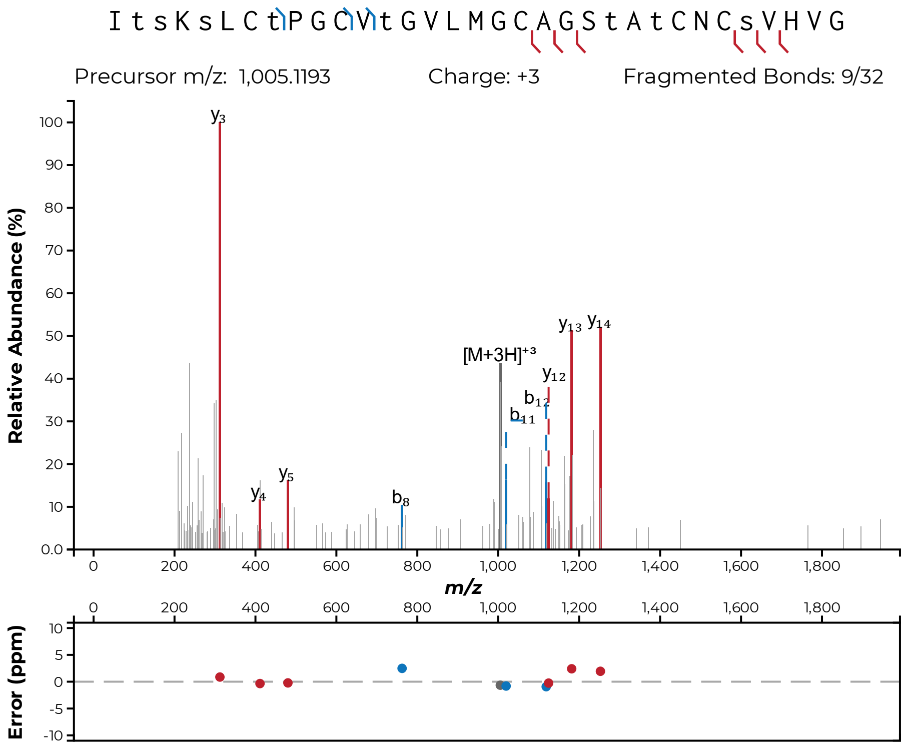
I t s K s L C t P G C V t G V L M G C A G S t A t C N C s V H V G
Precursor m/z: 1,005.1193
Charge: +3
Fragmented Bonds: 9/32
Relative Abundance (%)
m/z
Error (ppm)
[M+3H]+3
y3
y4
y5
b8
b11
b12
y12
y13
y14

B

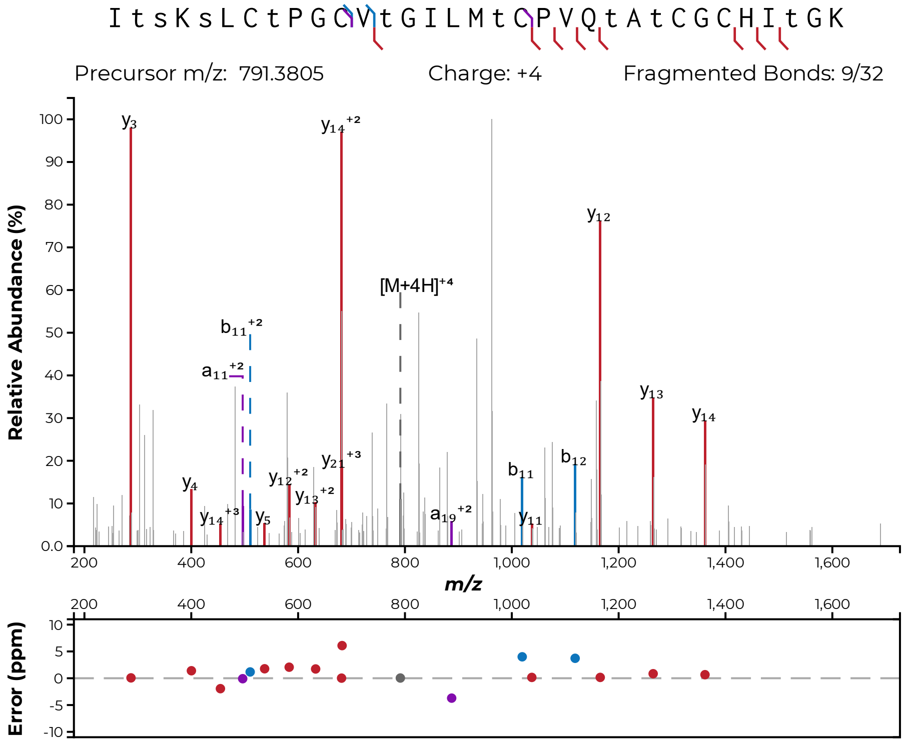
I t s K s L C t P G C V t G I L M t C P V Q t A t C G C H I t G K
Precursor m/z: 791.3805
Charge: +4
Fragmented Bonds: 9/32
Relative Abundance (%)
m/z
Error (ppm)
[M+4H]+4
y3
y4
y14+3
y5
b11+2
a11+2
y12+2
y13+2
y21+3
y14+2
a19+2
b11
y11
b12
y12
y13
y14

C

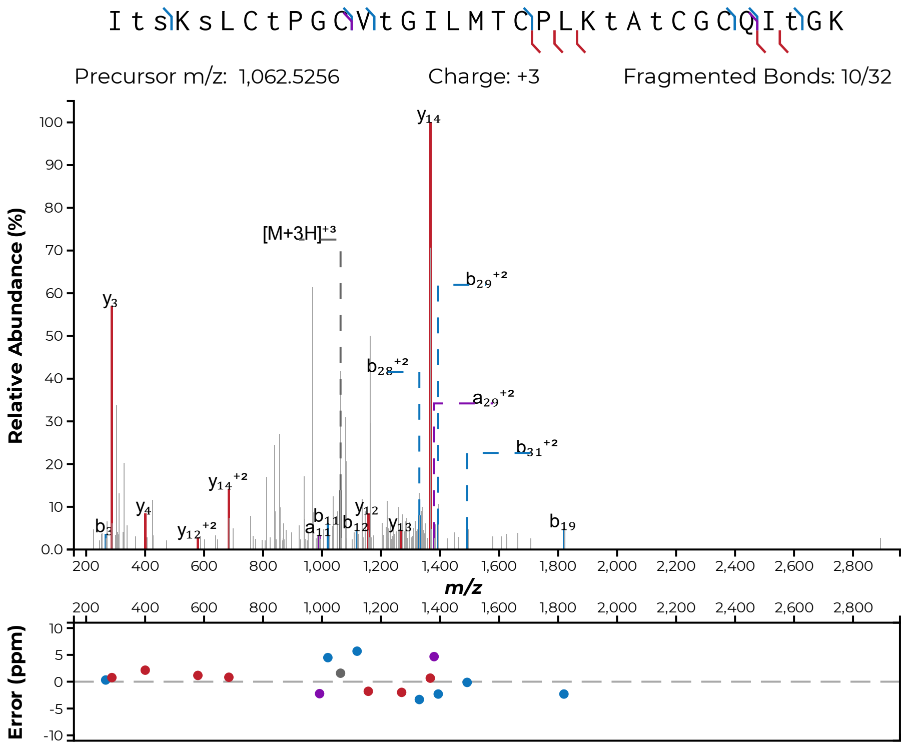
I t s K s L C t P G C V t G I L M T C P L K t A t C G C Q I t G K
Precursor m/z: 1,062.5256
Charge: +3
Fragmented Bonds: 10/32
Relative Abundance (%)
m/z
Error (ppm)
[M+3H]+3
y14
y3
b29+2
b28+2
a29+2
b31+2
y14+2
y4
y12+2
y12
b11
a11
b12
y13
b3
b19

D

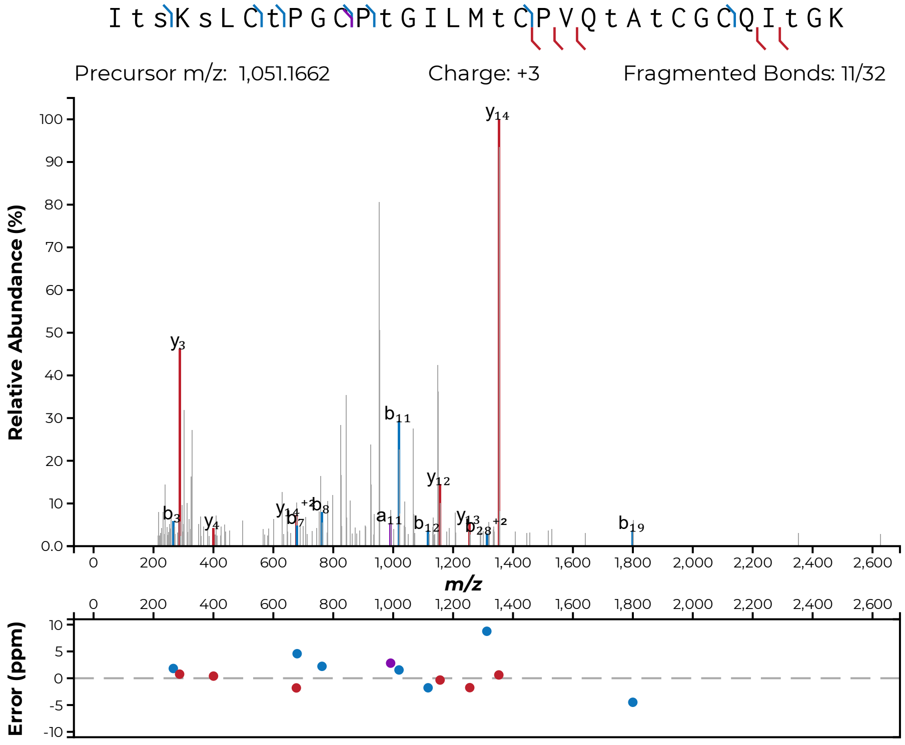
I t s K s L C t P G C P t G I L M t C P V Q t A t C G C Q I t G K
Precursor m/z: 1,051.1662
Charge: +3
Fragmented Bonds: 11/32
Relative Abundance (%)
m/z
Error (ppm)
y14
y3
b11
y12
b3
y4
b7
b8
a11
b12
y13
b28+2
b19

E

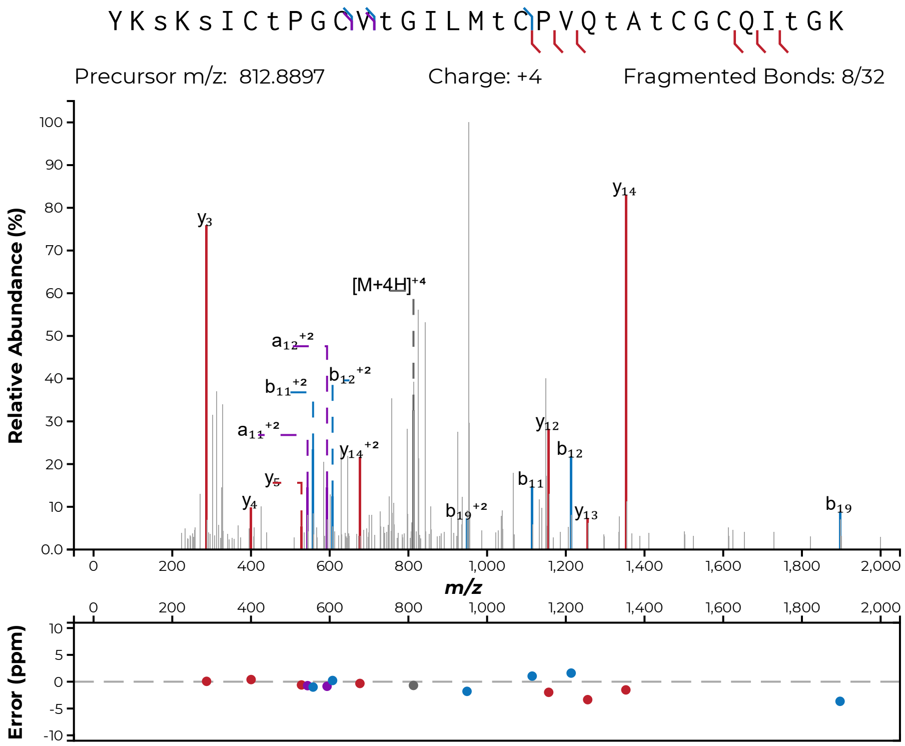
Y K s K s I C t P G C V t G I L M t C P V Q t A t C G C Q I t G K
Precursor m/z: 812.8897
Charge: +4
Fragmented Bonds: 8/32
Relative Abundance (%)
m/z
Error (ppm)
[M+4H]+4
y3
y4
y5
y12
y13
y14
b11
b12
b19
a11+2
a12+2
b11+2
b12+2
y14+2
b19+2

F

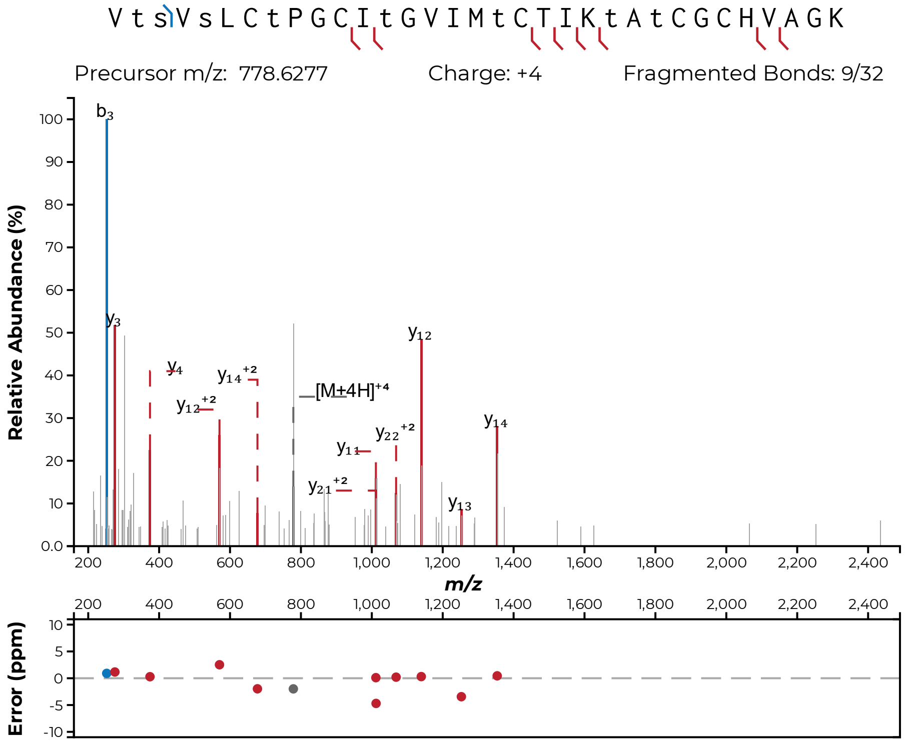
V t s V s L C t P G C I t G V I M t C T I K t A t C G C H V A G K
Precursor m/z: 778.6277
Charge: +4
Fragmented Bonds: 9/32
Relative Abundance (%)
m/z
Error (ppm)
[M+4H]+4
b3
y3
y4
y11
y12
y13
y14
y12+2
y14+2
y21+2
y22+2

G

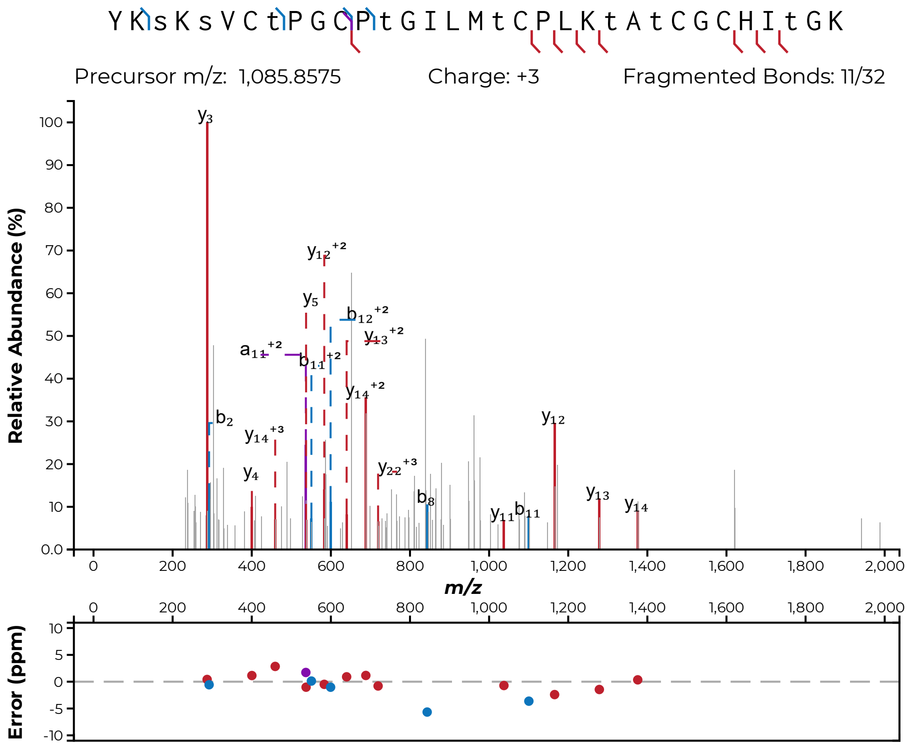
Y K s K s V C t P G C P t G I L M t C P L K t A t C G C H I t G K
Precursor m/z: 1,085.8575
Charge: +3
Fragmented Bonds: 11/32
Relative Abundance (%)
m/z
Error (ppm)

H

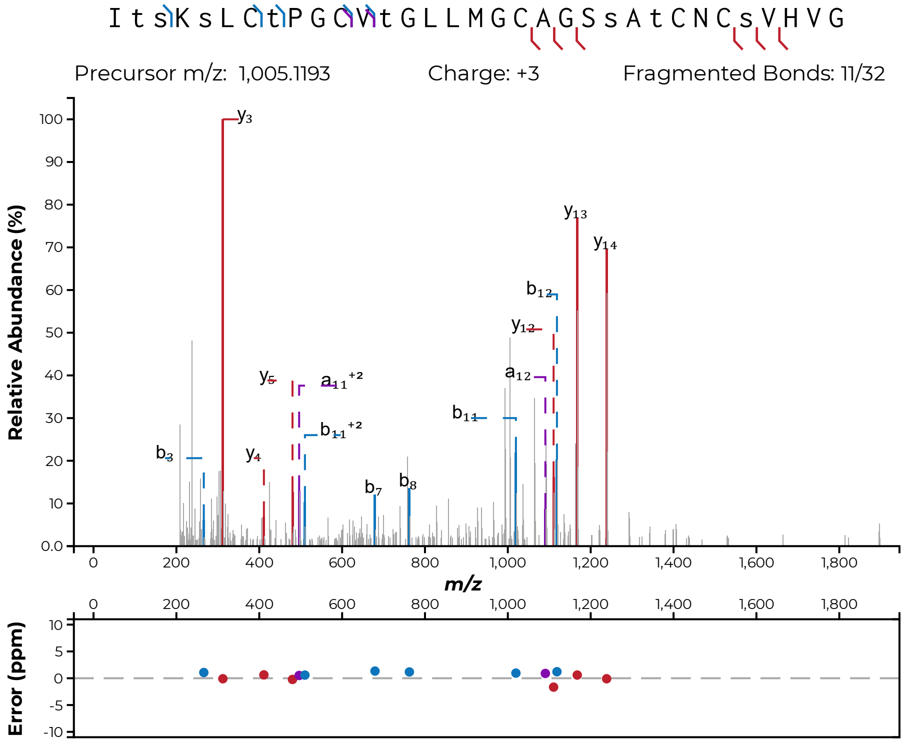
I t s K s L C t P G C V t G L L M G C A G S s A t C N C s V H V G
Precursor m/z: 1,005.1193
Charge: +3
Fragmented Bonds: 11/32
Relative Abundance (%)
m/z
Error (ppm)

I

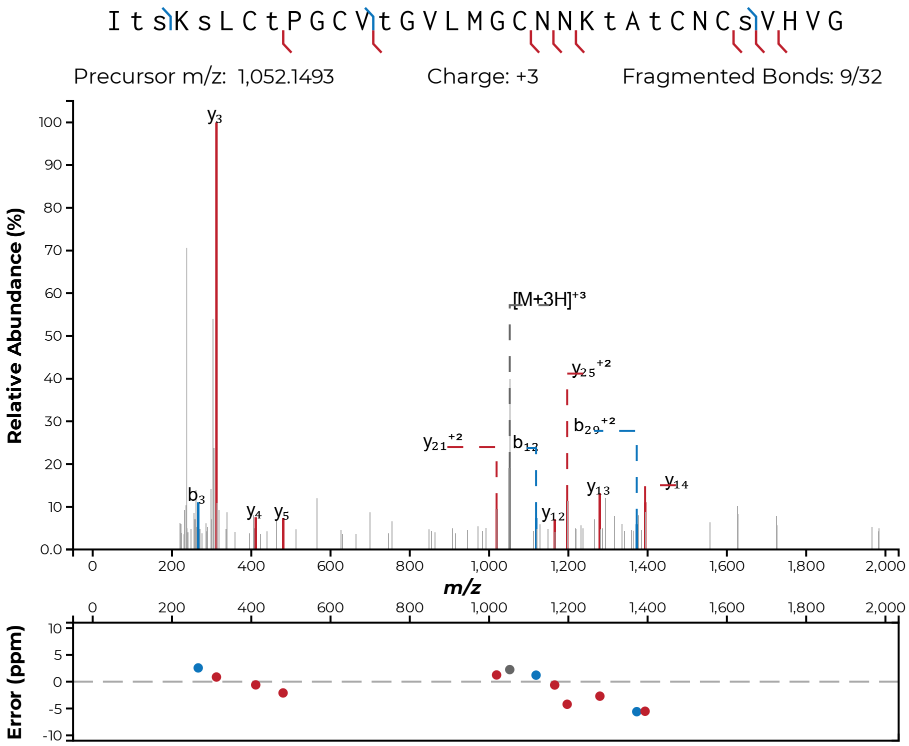

J

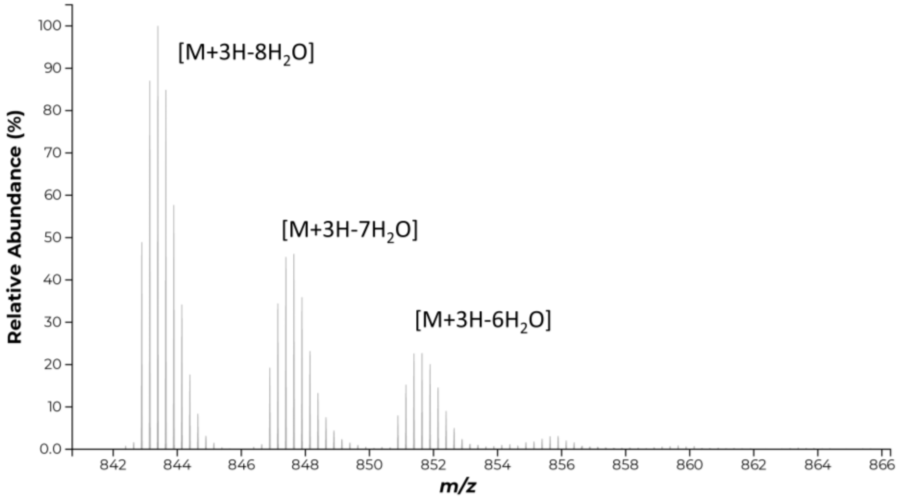

Fig. S6. Tandem MS analysis of lantibiotics produced in *E. coli.* (A) Analog5 (B) Analog4 (C) Analog3 (D) Analog2 (E) Analog1 (F) Lan-CE02 (G) Lan-Df (H) Lan-P49.1 (I) Lan-P49.2. For all peptides, only the peptide with the highest number of dehydrations was analyzed. For all lantibiotics, fragment ions are seen in the hinge region, at the linear C-terminal tail peptide, and between the B and C-rings. Sometimes fragmentation is also observed between the A and B rings. Generally, no fragmentation is detected inside rings with the exception of fragments that have also been reported for nisin (e.g. Fig. S6 in ref [10]): low intensity ions in the A-ring and before the Pro in the B-ring. The fragmentation patterns are very similar for all peptides and strongly resemble the fragmentation observed for nisin, thus providing strong support that the peptides all possess the same ring pattern. Below each MS/MS spectrum, a graph of the ppm

errors between calculated and observed m/z values for each identified ion is shown.[11] Panel J is a portion of a high resolution ESI mass spectrum collected on a sample of nisin A from a commercial source. The mass range was adjusted to focus on the triply charged states (M+3H). The spectrum clearly shows that the sample contains significant quantities of fully dehydrated nisin (-8 H_2O) and partially dehydrated nisin (-7 H_2O and -6 H_2O).

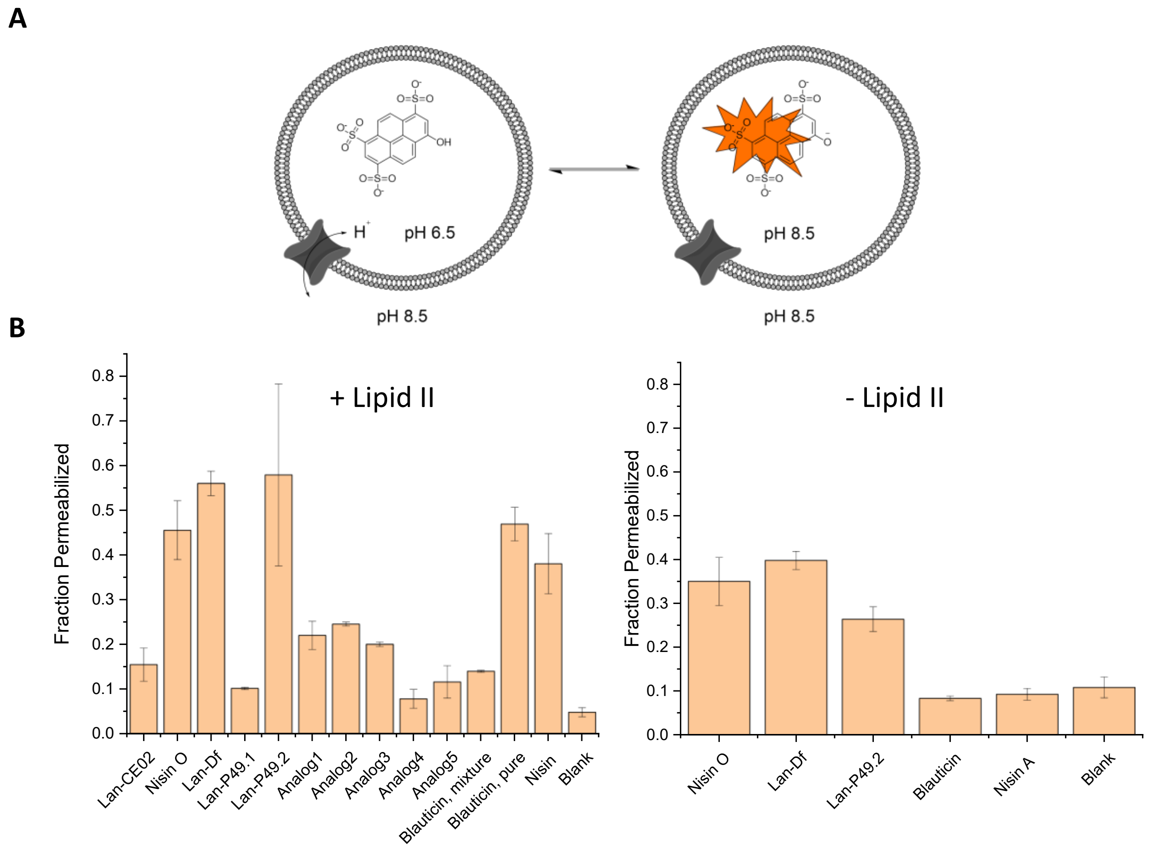

Fig. S7 Liposome permeabilization assays of nisin-like lantibiotics. (A) Scheme of liposome permeabilization assay. Pyranine encapsulated in liposomes containing low pH buffer exhibits low fluorescence. Fluorescence increases upon liposome permeabilization and pH increase. **(B)** Fraction of liposomes permeabilized by lantibiotics (375 nM). Liposomes were composed of DOPC with or without 0.1 mol% lipid II. Error bars represent the standard of deviation of three replicates.

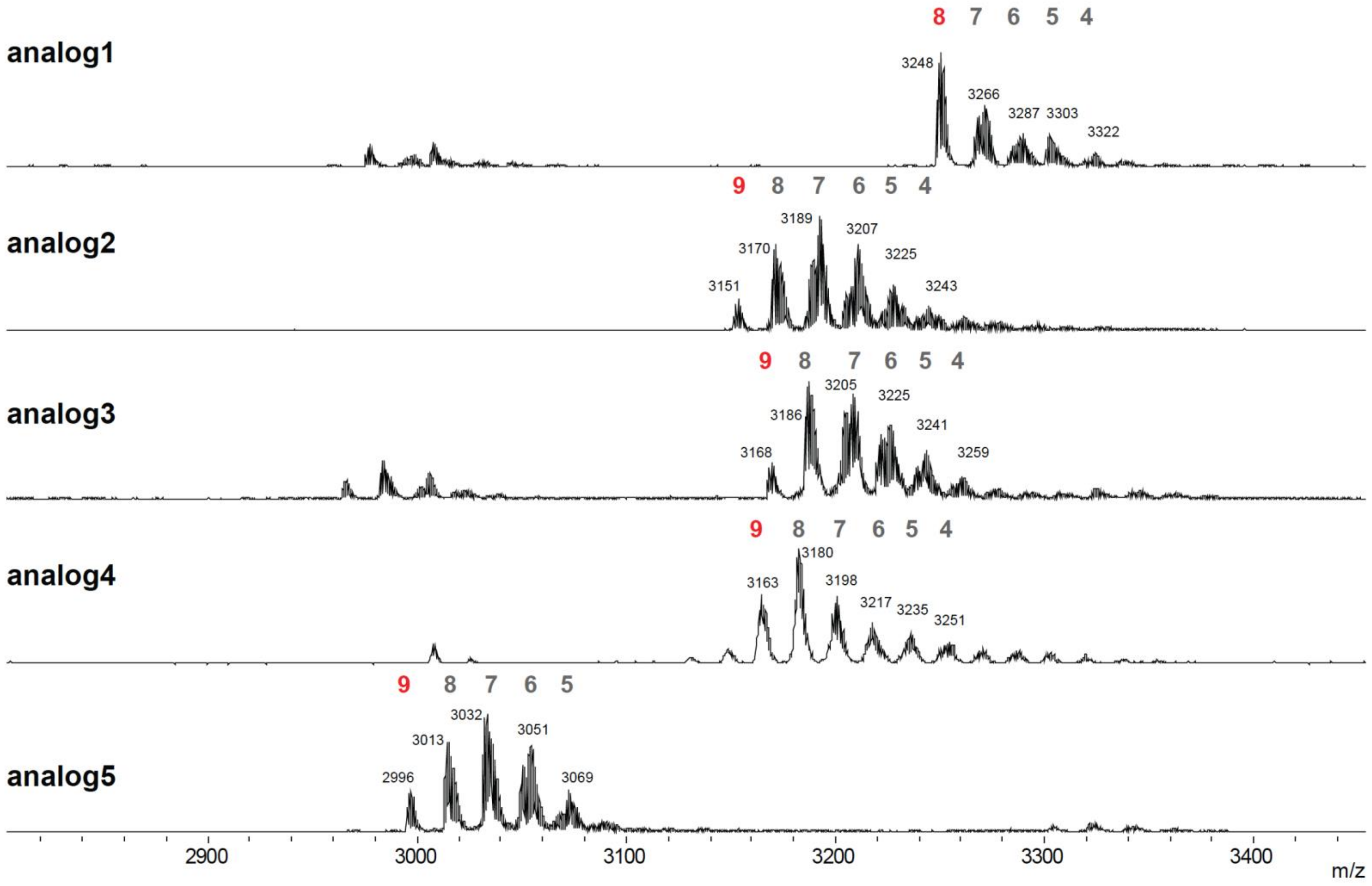

Fig. S8 MALDI-TOF mass spectra of purified blauticin variants. The numbers on top of the peaks indicate dehydration states for each peptide. Red numbers indicate fully dehydrated forms. Observed masses for each peak are labeled on top. Observed and expected masses for each compound are listed in Table S1.

Table S1. Lanthipeptide expected and observed masses (Da).*

Number of dehydrations	0	1	2	3	4	5	6	7	8	9
**Blauticin (expected)**	3316	3298	3280	3262	3244	3226	3208	3190	3172	3154
**Blauticin (observed)**	NO	NO	NO	NO	3245	3228	3210	3192	3175	3156
**Nisin O (expected)**	3373	3355	3337	3319	3301	3283	3265	3247	3229	NA
**Nisin O (observed)**	NO	NO	NO	NO	NO	3283	3267	3248	3228	NA
**Lan-Df (expected)**	3401	3383	3365	3347	3329	3311	3293	3275	3257	NA
**Lan-Df (observed)**	NO	NO	NO	NO	NO	NO	3294	3275	3257	NA
**Lan-CE02 (expected)**	3257	3239	3221	3203	3185	3167	3149	3131	3113	NA
**Lan-CE02 (observed)**	NO	NO	NO	NO	NO	3171	3151	3133	3113	NA
**Lan-P49.1 (expected)**	3159	3141	3123	3105	3087	3069	3051	3033	3015	NA
**Lan-P49.1 (observed)**	NO	NO	NO	3105	3087	3069	3051	3028	3014	NA
**Lan-P49.2 (expected)**	3300	3282	3264	3246	3228	3210	3192	3174	3156	NA
**Lan-P49.2 (observed)**	NO	NO	NO	NO	NO	3210	3193	3173	3155	NA
**analog1 (expected)**	3393	3375	3357	3339	3321	3303	3285	3267	3249	NA
**analog1 (observed)**	NO	NO	NO	3336	3322	3303	3287	3266	3248	NA
**analog2 (expected)**	3314	3296	3278	3260	3242	3224	3205	3187	3169	3151
**analog2 (observed)**	NO	NO	NO	3261	3243	3225	3207	3189	3170	3151
**analog3 (expected)**	3330	3312	3294	3276	3258	3240	3222	3204	3186	3168
**analog3 (observed)**	NO	NO	NO	3275	3259	3241	3225	3205	3186	3168
**analog4 (expected)**	3325	3307	3289	3271	3253	3235	3217	3198	3180	3162
**analog4 (observed)**	NO	3305	3287	3270	3251	3235	3217	3198	3180	3163
**analog5 (expected)**	3157	3139	3121	3103	3085	3067	3049	3031	3013	2995
**analog5 (observed)**	NO	NO	NO	NO	NO	3069	3051	3032	3013	2996

(*All masses are in Dalton; NA = not applicable; NO = not observed).

Table S2. List of oligonucleotides and gene fragments used in this study.

Name	**5' Sequence 3'**
pEVOL_GluRS1 (BPscsk)_gBlock	CCCGTTTTTTTGGGCTAACAGGAGGAATTAGATCTATGTCCACAGTGCGCACCCGTTTTGCTCCGTCTCCGACGGGTCG<br>TATGCATGTAGGAAATCTGCGTACCGCACTGTACGCATATTTAATTGCCAAACACGAAAACGGCTCATTCATGCTGCGT<br>ATTGAAGATACTGATCAAGAACGTTTTGTAGACGGTGCCCTGGAAATCATTTATCGCACGTTAGCCAAGACGGGCTTGA<br>TTCATGACGAGGGCCCGGATAAAGATGGAGGCTATGGTCCGTATGTACAGAGCGAACGTAATGCACAAGGCATTTATCT<br>CAACTACGCCAAACAACTGATTGAACAGGGTGATGCCTACTATTGCTTTTGTACGAAGGAACGGTTAGATTCCCTGAAG<br>GCGTCTGTAGGAGAAGATGGTAAAGAAATTGCTGTGTATGATAAACACTGCTTACATCTTTCTCGTGAGGAGGTGGAGG<br>CGAAACTGGCGGCCGGCGAACCGCATGTGATCCGTTTTAATATGCCAACGGAAGGGAACACCACCTTTCATGATGATAT<br>CTACGGAGACATCACGGTGCCGAATAATGAGCTGGACGACCTTATTCTGATCAAATCGGATGGTTACCCGACTTATAAC<br>TTTGCTAACGTCATTGATGATCACTTGATGGGTATCACGCACGTTGTGCGCGGCAATGAATATCTTAGCTCTAGCCCAA<br>AATATAATCGGATTTACGAAGCCTTTGGTTGGGAAATCCCGACATATGTGCACTGCCCGTTGATTACCAACGAAGAGCA<br>TAAGAAACTGTCCAAGCGTAGCGGGCACTCATCTTATGAAGACTTAACGGACCAAGGCTTCCTCACCGAAGCCATCGTT<br>AATTACGTAGCTCTGCTCGGGTGGTGCCCGGAGGATAATCGCGAGATCTTCAGTCTGGAAGAACTGGTGAAAGAGTTTG<br>ATTATCACCATATGAGTAAATCCCCGGCCGTCTTTGATATGACCAAGCTGAAGTGGATGAACGGGGAATATATTAAAGC<br>CATGGATTTTGATAAATTTTGTGATATGGCCCTGCCTTTCGTGAAAGAAGCGGTTAAAAGTGATCTGGATCTGAAAAAA<br>ATCGTCCAGATGGTGAAAACGCGCATTGAAGTCTTCCCCGATATTCCAGCCCTTATCGATTTCTTTGAAGAAGTGCCGG<br>AATACGATGTTTCTATGTATACCCATAAAAAAATGAAAACTAACCCGGAACTCTCCTTGGAGGTTCTGAAAAAAATCCT<br>GCCAGTACTGGAAGATTTTGAGGACTATACCAATGATGCTTTATATGATTTACTGTGTGGCTTTGCGAAAGAGAACGGC<br>TATAAGAACGGTCAGATCCTGTGGCCGATTCGTACAGCGCTGAGCGGGAAGCAGATGACTCCAGCGGGAGCTACCGAGA<br>TCTTGGAGGTGCTGGGTAAAGAAGAATCGATGAAGCGTCTTCATGCGGCCGTGGAAAAACTGGAATGCGTTTGAGTCGA<br>CCATCATCATCATCATCATTGAGTTTAAACGGTCTCCAGCTTGGCTGTTTTGGCGGATGAGAGAAGATTTTCAGCCTGA<br>TACAGATTAAATCAGAACGCAGAAGCGGTCTGATAAAACAGAATTTGCCTGGCGGCAGTAGCGCGGTGGTCCCACCTGA<br>CCCCATGCCGAACTCAGAAGTGAAACGCCGTAGCGCCGATGG
pEVOL_GluRS2 (BPscsk)_gBlock	CAGAAGTGAAACGCCGTAGCGCCGATGGTAGTGTGGGGTCTCCCCATGCGAGAGTAGGGAACTGCCAGGCATCAAATAA<br>AACGAAAGGCTCAGTCGAAAGACTGGGCCTTGTTTGTGAGCTCCCGGTCATCAATCATCCCCATAATCCTTGTTAGATT<br>ATCAATTTTAAAAAACTAACAGTTGTCAGCCTGTCCCGCTTTAATATCATACGCCGTTATACGTTGTTTACGCTTTGAG<br>GAATCCCATATGTCCACAGTGCGCACCCGTTTTGCTCCGTCTCCGACGGGTCGTATGCATGTAGGAAATCTGCGTACCG<br>CACTGTACGCATATTTAATTGCCAAACACGAAAACGGCTCATTCATGCTGCGTATTGAAGATACTGATCAAGAACGTTT<br>TGTAGACGGTGCCCTGGAAATCATTTATCGCACGTTAGCCAAGACGGGCTTGATTCATGACGAGGGCCCGGATAAAGAT<br>GGAGGCTATGGTCCGTATGTACAGAGCGAACGTAATGCACAAGGCATTTATCTCAACTACGCCAAACAACTGATTGAAC<br>AGGGTGATGCCTACTATTGCTTTTGTACGAAGGAACGGTTAGATTCCCTGAAGGCGTCTGTAGGAGAAGATGGTAAAGA<br>AATTGCTGTGTATGATAAACACTGCTTACATCTTTCTCGTGAGGAGGTGGAGGCGAAACTGGCGGCCGGCGAACCGCAT<br>GTGATCCGTTTTAATATGCCAACGGAAGGGAACACCACCTTTCATGATGATATCTACGGAGACATCACGGTGCCGAATA<br>ATGAGCTGGACGACCTTATTCTGATCAAATCGGATGGTTACCCGACTTATAACTTTGCTAACGTCATTGATGATCACTT<br>GATGGGTATCACGCACGTTGTGCGCGGCAATGAATATCTTAGCTCTAGCCCAAAATATAATCGGATTTACGAAGCCTTT

	GGTTGGGAAATCCCGACATATGTGCACTGCCCGTTGATTACCAACGAAGAGCATAAGAAACTGTCCAAGCGTAGCGGGC<br>ACTCATCTTATGAAGACTTAACGGACCAAGGCTTCCTCACCGAAGCCATCGTTAATTACGTAGCTCTGCTCGGGTGGTG<br>CCCGGAGGATAATCGCGAGATCTTCAGTCTGGAAGAACTGGTGAAAGAGTTTGATTATCACCATATGAGTAAATCCCCG<br>GCCGTCTTTGATATGACCAAGCTGAAGTGGATGAACGGGGAATATATTAAAGCCATGGATTTTGATAAATTTTGTGATA<br>TGGCCCTGCCTTTCGTGAAAGAAGCGGTTAAAAGTGATCTGGATCTGAAAAAAATCGTCCAGATGGTGAAAACGCGCAT<br>TGAAGTCTTCCCCGATATTCCAGCCCTTATCGATTTCTTTGAAGAAGTGCCGGAATACGATGTTTCTATGTATACCCAT<br>AAAAAAATGAAAACTAACCCGGAACTCTCCTTGGAGGTTCTGAAAAAAATCCTGCCAGTACTGGAAGATTTTGAGGACT<br>ATACCAATGATGCTTTATATGATTTACTGTGTGGCTTTGCGAAAGAGAACGGCTATAAGAACGGTCAGATCCTGTGGCC<br>GATTCGTACAGCGCTGAGCGGGAAGCAGATGACTCCAGCGGGAGCTACCGAGATCTTGGAGGTGCTGGGTAAAGAAGAA<br>TCGATGAAGCGTCTTCATGCGGCCGTGGAAAAACTGGAATGCGTTTGACTGCAGTTTCAAACGCTAAATTGCCTGATGC<br>GCTAC
pEVOL_CUCtRNA(BPscsk)_gBlock	GAATGCGTTTGACTGCAGTTTCAAACGCTAAATTGCCTGATGCGCTACGCTTATCAGGCCTACATGATCTCTGCAATAT<br>ATTGAGTTTGCGTGCTTTTGTAGGCCGGATAAGGCGTTCACGCCGCATCCGGCAAGAAACAGCAAACAATCCAAAACGC<br>CGCGTTCAGCGGCGTTTTTTCTGCTTTTCTTCGCGAATTAATTCCGCTTCGCAACATGTGAGCACCGGTTTATTGACTA<br>CCGGAAGCAGTGTGACCGTGTGCTTCTCAAATGCCTGAGGCCAGTTTGCTCAGGCTCTCCCCGTGGAGGTAATAATTGA<br>CGATATGATCAGTGCACGGCTAACTAAGCGGCCTGCTGACTTTCTCGCCGATCAAAAGGCATTTTGCTATTAAGGGATT<br>GACGAGGGCGTATCTGCGCAGTAAGATGCGCCCCGCATTGGTTCGTTGGTCAAGCGGTtAAGACGCCGCCCTCTCACGG<br>CGGAAaCAGGGGTTCGATTCCCCTACGAACTGCAATTCGAAAAGCCTGCTCAACGAGCAGGCTTTTTTGCATGCTCGAG<br>CAGCTCAGGGTCGAATTTGCTTTCGAATTTCTGCCATTCATCCGCTTATTATCACTTATTCAGGCGTAGCAAC
pEVOL_UUCtRNA(BPscsk)_gBlock	GCAGTTTCAAACGCTAAATTGCCTGATGCGCTACGCTTATCAGGCCTACATGATCTCTGCAATATATTGAGTTTGCGTG<br>CTTTTGTAGGCCGGATAAGGCGTTCACGCCGCATCCGGCAAGAAACAGCAAACAATCCAAAACGCCGCGTTCAGCGGCG<br>TTTTTTCTGCTTTTCTTCGCGAATTAATTCCGCTTCGCAACATGTGAGCACCGGTTTATTGACTACCGGAAGCAGTGTG<br>ACCGTGTGCTTCTCAAATGCCTGAGGCCAGTTTGCTCAGGCTCTCCCCGTGGAGGTAATAATTGACGATATGATCAGTG<br>CACGGCTAACTAAGCGGCCTGCTGACTTTCTCGCCGATCAAAAGGCATTTTGCTATTAAGGGATTGACGAGGGCGTATC<br>TGCGCAGTAAGATGCGCCCCGCATTGGCTCCATGGTCAAGCGGTtAAGACACCGCCCTTTCACGGCGGTAaCAGGGGTT<br>CAAATCCCCTTGGAGTCACAATTCGAAAAGCCTGCTCAACGAGCAGGCTTTTTTGCATGCTCGAGCAGCTCAGGGTCGA<br>ATTTGCTTTCGAATTTCTGCCATTCATCCGCTTATTATCACTTATTCAGGCGTAGCAACCAGGCGTTTAAGGGCACCAA<br>TAACTGCCTTAAAAAAATTACGCCCCGCCCTGCCACTCATCGCAGTACTGTTGTAATTCATTAAGCATTCTGCCGACAT<br>GGAAGCCATCACAAACGGCATGATGAACCTGAATC
pEVOL_GluRS1(BPscsk)F	CGTTTTTTTGGGCTAACAGGAG
pEVOL_GluRS1(BPscsk)R	GCGCTACGGCGTTTC
pEVOL_GluRS2(BPscsk)F	CAGAAGTGAAACGCCGTAG
pEVOL_GluRS2(BPscsk)R	GTAGCGCATCAGGCAATTTAG
pEVOL_BB_F	CTCGAGCAGCTCAGG
pEVOL_BB_R	GAGACGGAGCAAAACGGGTGCGCACTGTGGACATAGATCTAATTCCTCCTGTTAGC

pEVOL_CUCtRNA(BPscsk)F	GAATGCGTTTGACTGCAGTTTCAAACGCTAAATTGCCTGA
pEVOL_CUCtRNA(BPscsk)R	GTTGCTACGCCTGAATAAGTGATAATAAGCGGATGAATG
pEVOL_UUCtRNA(BPscsk)F	GCAGTTTCAAACGCTAAATTG
pEVOL_UUCtRNA(BPscsk)R	GATTCAGGTTCATCATGCC
**Blauticin Analogs**	
pETDuet_nisin O_gBlock	ATAACAATTCCCCTCTAGAAATAATTTTGTTTAACTTTAAGAAGGAGATATACCATGGGCAGCAGCCATCACCATCATCACCACAGCCAGGATCCGAATTCGAGCTCGGCGCGCCTGCAGGTCGACATGGCGAAATTCGACGACTTCGATCTGGACGTTACCAAAACCGCAGCTGGCGAAGGCGGTGTTGAACCTCGTTATAAAGTAAATCTGCTTGTACGCCAGGCTGCCCGACCGGCATTCTGATGACTTGCCCACTGAAAACGGCAACCTGTGGATGTCATATTACCGGCAAATAA
pETDuet_D. formicigenerans_gBlock	ATAACAATTCCCCTCTAGAAATAATTTTGTTTAACTTTAAGAAGGAGATATACCATGGGCAGCAGCCATCACCATCATCACCACAGCCAGGATCCGAATTCGAGCTCGGCGCGCCTGCAGGTCGACATGGCGAAATTCGACGACTTCGATCTGGACGTTACCAAAACCGCAGCTGGCGAAGGCGGTGTTGAACCGCGTTATAAATCCAAAAGCGTGTGCACCCCGGGCTGTCCGACAGGTATTCTGATGACCTGCCCGCTGAAAACCGCCACCTGTGGGTGTCATATTACCGGCAAGTAA
pETDuet_Clostridum sp E02_gBlock	ATAACAATTCCCCTCTAGAAATAATTTTGTTTAACTTTAAGAAGGAGATATACCATGGGCAGCAGCCATCACCATCATCACCACAGCCAGGATCCGAATTCGAGCTCGGCGCGCCTGCAGGTCGACATGGCGAAATTCGACGACTTCGATCTGGACGTTACCAAAACCGCAGCTGGCGAAGGCGGTGTTGAACCGCGTGTGACGTCCGTGTCCTTATGTACGCCGGGATGCATCACGGGCGTAATCATGACTTGTACCATTAAAACGGCAACCTGTGGCTGCCACGTGGCCGGCAAATAA
pETDuet_Pseudobutyrivibrio_sp49_1_gBlock	ATAACAATTCCCCTCTAGAAATAATTTTGTTTAACTTTAAGAAGGAGATATACCATGGGCAGCAGCCATCACCATCATCACCACAGCCAGGATCCGAATTCGAGCTCGGCGCGCCTGCAGGTCGACATGGCGAAATTCGACGACTTCGATCTGGACGTTACCAAAACCGCAGCTGGCGAAGGCGGTGTTGAACCGCGCATCACTTCGAAATCTCTGTGTACCCCGGGTTGCGTCACCGGGCTGCTTATGGGTTGTGCCGGCAGCTCTGCCACATGTAATTGTAGTGTGCATGTTGGCTAA
pETDuet_Pseudobutyrivibrio_sp49_2_gBlock	ATAACAATTCCCCTCTAGAAATAATTTTGTTTAACTTTAAGAAGGAGATATACCATGGGCAGCAGCCATCACCATCATCACCACAGCCAGGATCCGAATTCGAGCTCGGCGCGCCTGCAGGTCGACATGGCGAAATTCGACGACTTCGATCTGGACGTTACCAAAACCGCAGCTGGCGAAGGCGGTGTTGAACCGCGCATCACATCGAAATCGCTGTGCACTCCAGGTTGCGTGACCGGTGTACTGATGGGGTGTAACAACAAAACCGCAACTTGTAATTGCAGTGTCCACGTCGGATGA
pETDuet_BB_F	CATAATGCTTAAGTCGAACAGAAAG
pETDuet_BB_R	CAAAATTATTTCTAGAGGGGAATTGTTAT
pETDuet_nisin_DF_CE02_sp49_F	GAATTGTGAGCGGATAACAATTCCCCTCTAGAAATAATTTTG
pETDuet_nisin O_R	TGTTCGACTTAAGCATTATGCGGCCGCTTATTTGCCGGTAATATGACATCC

pETDuet_D. formicigenerans_R	TGTTCGACTTAAGCATTATGCGGCCGCTTACTTGCCGGTAATATGACAC
pETDuet_Clostridum sp E02_R	TGTTCGACTTAAGCATTATGCGGCCGCTTATTTGCCGGCCACG
pETDuet_Pseudobutyrivibrio_sp49_1_R	TGTTCGACTTAAGCATTATGCGGCCGCTTAGCCAACATGCACACTAC
pETDuet_Pseudobutyrivibrio_sp49_2_R	TGTTCGACTTAAGCATTATGCGGCCGCTCATCCGACGTGGAC
**Blauticin analogs**	
pETDuet_SAR1_ITtoYK_gBlock	ATGGGCAGCAGCCATCACCATCATCACCACAGCCAGGATCCGAATTCGAGCTCGGCGCGCCTGCAGGTCGACATGGCGAAATTCGACGACTTCGATCTGGACGTTACCAAAACCGCAGCTGGCGAAGGCGGTGTTGAACCGCGTTATAAATCCAAAAGCCTGTGTACCCCGGGTTGCGTTACCGGTATTCTGATGACCTGTCCGGTTCAAACCGCTACCTGTGGTTGTCAGATCACCGGCAAATAA
pETDuet_SAR2_V12P_gBlock	ATGGGCAGCAGCCATCACCATCATCACCACAGCCAGGATCCGAATTCGAGCTCGGCGCGCCTGCAGGTCGACATGGCGAAATTCGACGACTTCGATCTGGACGTTACCAAAACCGCAGCTGGCGAAGGCGGTGTTGAACCGCGTATTACCTCCAAAAGCCTGTGTACCCCGGGTTGCCCGACCGGTATTCTGATGACCTGTCCGGTTCAAACCGCTACCTGTGGTTGTCAGATCACCGGCAAATAA
pETDuet_SAR3_VQtoLK_gBlock	ATGGGCAGCAGCCATCACCATCATCACCACAGCCAGGATCCGAATTCGAGCTCGGCGCGCCTGCAGGTCGACATGGCGAAATTCGACGACTTCGATCTGGACGTTACCAAAACCGCAGCTGGCGAAGGCGGTGTTGAACCGCGTATTACCTCCAAAAGCCTGTGTACCCCGGGTTGCGTTACCGGTATTCTGATGACCTGTCCGCTGAAAACCGCTACCTGTGGTTGTCAGATCACCGGCAAATAA
pETDuet_SAR4_Q29H_gBlock	ATGGGCAGCAGCCATCACCATCATCACCACAGCCAGGATCCGAATTCGAGCTCGGCGCGCCTGCAGGTCGACATGGCGAAATTCGACGACTTCGATCTGGACGTTACCAAAACCGCAGCTGGCGAAGGCGGTGTTGAACCGCGTATTACCTCCAAAAGCCTGTGTACCCCGGGTTGCGTTACCGGTATTCTGATGACCTGTCCGGTTCAAACCGCTACCTGTGGTTGTCATATCACCGGCAAATAA
pETDuet_SAR5_sp49_2_AGS_gBlock	ATGGGCAGCAGCCATCACCATCATCACCACAGCCAGGATCCGAATTCGAGCTCGGCGCGCCTGCAGGTCGACATGGCGAAATTCGACGACTTCGATCTGGACGTTACCAAAACCGCAGCTGGCGAAGGCGGTGTTGAACCGCGTATCACATCGAAATCGCTGTGCACTCCAGGTTGCGTGACCGGTGTACTGATGGGGTGTGCCGGCAGCACCGCAACTTGTAATTGCAGTGTCCACGTCGGATGA
pETDuerSAR_BB_F	CCGCATAATGCTTAAGT
pETDuerSAR_BB_R	CCATGGTATATCTCCTTC
pETDuerSAR_SAR12345_F	GAAGGAGATATACCATGGGCAGCAGC
pETDuerSAR_SAR123_R	CATTATGCGGCCGCTTATTTGCCGGTGATCTG

pETDuerSAR_SAR4_R	CATTATGCGGCCGCTTATTTGCCGGTGATATGAC
pETDuerSAR_SAR5_R	CATTATGCGGCCGCTCATCCGACGTGGAC

Table S3. List of bacterial genomes and PFAM HMM used in this study.

**Pathogens**	**NCBI Accession**
Enterococcus faecium ATCC700221	GCF_009734005.1
Staphylococcus aureus USA300	GCF_017834975.1
Staphylococcus epidermidis SK135	GCF_000177115.1
Listeria monocytogenes 10403S	GCF_000168695.2
Clostridioides difficile VPI10463	GCA_001995155.1
**Commensals**	**NCBI Accession**
Anaerostipes hadrus MSK.14.23	GCF_013302595.1
Blautia obeum MSK.18.40	GCF_013299585.1
Blautia luti MSK.20.18	GCF_020553125.1
Erysipeloclostridium ramosum MSK.23.96	GCF_019125475.1
Dorea formicigenerans MSK.17.61	GCF_013300535.1
Coprococcus comes MSK.11.23	GCF_013301565.1
Coprococcus eutactus MSK.18.32	GCF_013299605.1
Faecalicatena fissicatena MSK.9.3	GCF_013300195.1
**Lantibiotic resistance gene**	**HMM/Uniprot accession**
**Cell wall biosynthesis**	
murG	Prokka annotation
murJ	Prokka annotation
**Penicillin-binding proteins**	Prokka annotation
dltA	TIGR01734.1
dltB	TIGR04091.1
dltD	PF04914
**Efflux pump**	
lanF	Prokka annotation

lanE	Prokka annotation
lanG	Prokka annotation
bceA	Prokka annotation
bceB	Prokka annotation
**Others**	
telA	PF05816
mprF	Q2G2M2; Q8Y6I9
nsr	P23648
lanI	PF18218

41 unique lanthipeptide candidates from the gut microbiome

>WP_065541929.1_1408289 Blautia coccoides
MAKFDDFDLDVTKTAAGEGGVEPRITSKSLCTPGCVTGILMTCPVQTATCGCQITGK
>WP_117638663.1_224961 Dorea formicigenerans
MAKFDDFDLDVTKTAAQGGIEPKYKSKSVCTPGCPTGILMTCPLKTATCGCHITGK
>WP_119197961.1_129175 Blautia sp. AM47-4
MAKFDDFDLDVTKTAAQGGIEPKYKSKSACTPGCPTGILMTCPLKTATCGCHITGK
>WP_119197961.1_129369 Blautia sp. AM47-4
MAKFDDFDLDVTKTAARGGIEPKYKSKSACTPGCPTGILMTCPLKTATCGCHITGK
>WP_117994378.1_29961 [Ruminococcus] gnavus
MPNFDDFDLDLRENGDDSVMQTGVSITSDGFCDTDVITSLVSCTRNGQFATCMCSTCCTGHTAPAGCSTGC
>WP_090169706.1_68663 Pseudobutyrivibrio sp. 49
MEKFNNDFNLDVTVSKDDTKKADVKITSKSLCTPGCVTGVLMGCNNKTATCNCSVHVG
>WP_070088801.1_6782 Merdimonas faecis
LCGLKNSATSKQTVIEGEKIMGKMDDFDLDLRKIAENGNSANALSASDMITSEIISKVTETITRTFKGQCVSVETPTTGMTSAC
CKKGGTDVEPQCVP
>WP_090169706.1_68164 Pseudobutyrivibrio sp. 49
MEKFNNDFNLDVTVSKDDTKKADVKITSKSLCTPGCVTGLLMGCAGSSATCNCSVHVG
>WP_090169706.1_68413 Pseudobutyrivibrio sp. 49
MEKFNNDFNLDVTVSKDDAKKADVKITSKSLCTPGCVTGLLMGCAGSSATCNCSVHVG
>WP_101696280.1_129779 Clostridium minihomine
MYHRRKNIMGKFDDFDLDFTKVSAAAENSSERAGIAVPKLAINWSKISCACSPSDMTVCRAGAPGQLRC

>WP_124067899.1_52626 Clostridium sp. E02
MAKFDDFDLDLKATSTKSGGISPMVTSVSLCTPGCITGVIMTCTIKTATCGCHVAGK
>WP_125152452.1_480876 Clostridium sp. Marseille-P4200
LVTTYLKNILEGGKMGKLNDFDLDLKVKETTKKGVEPTWKSKSFCTPGCVTGILMTCTSNGCK
>WP_065541929.1_1408112 Blautia coccoides
MAKFDDFDLDVTRMESGNNKTQPRITSISLCTSGCKTAFFGTCSFTKDK
>WP_068555846.1_216193 Thermotalea metallivorans
MPKYDDFDLDIQVKKVDNLVEPNITSVSLCTPGTCWNTCGGTSTLNTNCCIATYLCSLAESCR
>WP_070088808.1_6842 Merdimonas faecis
MGKMDDFDLDLRKIAENGNSANALSASDMITSEIISKVTETITRTFKGQCVSVETPTTGMTSACCKKGGTDVEPQCVP
>WP_008429197.1_38472 Clostridium sp. LS
MREKNMGKLDDFDLDVKVKADTTKVGPAITSKSLCTPGCITGVLMCITQNSCVSCKSCIKC
>WP_068555846.1_215943 Thermotalea metallivorans
MPKYDDFDLDIQVKEVSNAAEPNITSVSLCTPGTCYQGCSGDTTFNSNCCPSNYTVCITKTCFTCA
>WP_118445440.1_623 [Ruminococcus] gnavus
MNKDFDLNLKADKSATEGPAPRITSVAYCTPGCVTGELCGSSECGLTRNCTGSILWA
>WP_090169706.1_68906 Pseudobutyrivibrio sp. 49
MAKLKNDFDLDLTVSKDNTKSADVQFKSISLCTPGCPTGILMGCHKCPSGSDTVYTK
>WP_085321196.1_7739 Clostridium botulinum
MGKFDDFDLDVNVNKGKEKQSRIYSCTPGTCARTCDEACAPNYTFGTNCCASFNICFFQ
>WP_118445440.1_405 [Ruminococcus] gnavus
MNKDFDLNLKADKSTTEGPAPRITSVAYCTPGCITGDLCGSSECCLTRNCTGSILCA
>WP_118445440.1_1056 [Ruminococcus] gnavus
MNKDFDLNLKTDKNTTEGSAPRITSVAYCTPGCITGELCGSSECGLTRNCTGSILCA
>WP_118445440.1_810 [Ruminococcus] gnavus
MKTVMKGEKKMNKDFDLNLKADKSATEGPAPRITRVAYCTPGCVTGELCGSSECGLTRNCTGSILCA
>WP_077834666.1_106377 Clostridium roseum
VSSLSIIVNVNMSYLYILKQIIIKGDIIMPKFDDFDLDVKINKGKGVKPQIATSAVACTPGSCWGCPETSTFASACCNVSDNCG
D
>WP_077852909.1_9492 Clostridium aurantibutyricum
VSSLSIIVNVNISYLYILKQIIIKGDIIMPKFDDFDLDVKINKGKGVKPQIATSAVACTPGSCWGCPETSTFASACCNVSDNCGD
>WP_118704912.1_5418 Ruminococcus sp. AM49-10BH

MANYDDFDLDIRKIKGNMEAEPKGTVAICLTTGTATTCTMPTLCDTSIVTLASCDGTCVGCTNTEKPCSANTCSACNSYCGG
ACRK
>WP_118662284.1_8946 Coprobacillus sp. AF13-4LB
MNTLKDFDVNLNSVNNDDGDDTSTYSLWTSATTVPCSVAASISALSAFSSNWTVTGDPNYTESKC
>WP_080549726.1_102266 Clostridium perfringens
MLKDFDLNLKMETGSSDDKEPRITSVAYCTPGCLTGELCGSSECGLTRSCTNTWLCS
>WP_016224249.1_1916777 Lachnospiraceae bacterium 3-2
MSNFKDFDLDLRNVASGGNAEPNGTTLVCVASKMTIKQKCNSVETPTTGMTSACCKKKNAADEPQCV
>WP_087213613.1_6459 Drancourtella sp. An177
MGNLNDFDLDLRKFAQNTEGEGRVDTGDVVSITTSITTIPCQLLSLYSVEKCGEPSNAAIPTTAMTVNCCTKEGGNVPNCV
>WP_119245824.1_43152 Ruminococcus sp. AM50-15BH
MAKFDDFDLDIIEKKVRRDIIPASITSQHSFCTPNCLTGFLCPPKTQLTCTCKLKGQ
>WP_117416421.1_13043 Clostridium sp. PI-S10-A1B
MNKDFDLDLKMDKGSVDVGESKIASIVYCTSGCLTGTTCETSECGITRSCTNTVLCY
>WP_118627011.1_3447 Clostridium sp. AM43-3BH
MGKYDDFDLDLKKVMSNNTASEAESVVSILQTLATECFTFDGSNTCNCPSTDCSPCDMTTSCRRADPQIMRC
>WP_118470785.1_29014 Coprobacillus sp. AF19-3
MKDVKLDDFDLDIVGGDDNENDDGVSPASSISTGWVSISVWISNGLSTNFTESKC
>WP_066580644.1_3898242 Clostridium sp. Marseille-P2538
LQYGGKAMGKYDDFDLNVQDENIGGDSSGAQPKGFWSEVGKSVVSSLVTSCTGNCPSVKCTGNCTSTCTTSMTTNCLCHR
R
>WP_117908709.1_956 Ruminococcus sp. AF32-2AC
MANYDDFDLDIRKIKGNMEAEPKGTAAICLTTGTATTCTMPTLCDTSIVTLASCDGTCVGCTNTEKPCSANTCSACNSYCGG
ACRK
>WP_119623224.1_62755 Lachnospiraceae bacterium GAM79
MGNYSDFDLDIRSGEPAEPAAITSKACEVLWEMATASIDYCGKISELICPTDACTVGCSSDTCSACHSYCGAACRRIG
>WP_106062361.1_54850 Clostridium liquoris
MPNFEDFDLDLKKVKTNSDKVSVNTPTPTISYLVHCNTADCPSTDCSPGDMTIGCYAADKGVCRC
>WP_105309223.1_429064 Dorea sp. Marseille-P4042
MKRKELIMGKYDEFDLDIKEVKANSSKGTARSTWGCVEKSIEISQFVTNALSCQSCGVCSAGGRSQCAPCTGSSKGSQARC
>WP_005342481.1_22836 Dorea formicigenerans
MKRKELIMGKYDEFDLDIKEVKANSSKGTARSTWGCVEKSIQISQFVTNALSCQSCGVCSAGGRSQCAPCTGSSKGSQARC
>WP_107030381.1_23895 Coprobacillus sp. AM17-34

MPNLDEFDLDPVVSTAENGNDGISPQSYSVTTKPCLVTDVISVLSQVSTSLSTNPGYTESKC

Six unique class I lantibiotic sequences

>BpSCSK BpcA1 Blauticin
MAKFDDFDLDVTKTAAGEGGVEPRITSKSLCTPGCVTGILMTCPVQTATCGCQITGK
>Dorea formicigenerans Lan-Df
MAKFDDFDLDVTKTAAQGGIEPKYKSKSVCTPGCPTGILMTCPLKTATCGCHITGK
>Pseudobutyrivibrio sp. 49_2 Lan-P49.2
MEKFNNDFNLDVTVSKDDTKKADVKITSKSLCTPGCVTGVLMGCNNKTATCNCSVHVG
>Pseudobutyrivibrio sp. 49_1 Lan-P49.1
MEKFNNDFNLDVTVSKDDTKKADVKITSKSLCTPGCVTGLLMGCAGSSATCNCSVHVG
>Clostridium sp. E02 Lan-CE02
MAKFDDFDLDLKATSTKSGGISPMVTSVSLCTPGCITGVIMTCTIKTATCGCHVAGK
>Blautia obeum NsoA1 Nisin O
MGKFDDFDLDVTKTAAQGGIEPKYKSKSACTPGCPTGILMTCPLKTATCGCHITGK
>NisA Nisin A
MSTKDFNLDLVSVSKKDSGASPRITSISLCTPGCKTGALMGCNMKTATCHCSIHVSK

Codon optimized gene sequences for expression in *E. coli*

>BpcB, codon optimized
ATGAAGAAGCTGTTCTACGACATCGGCGAGTTCATGTATCGCCGTCCGACCGAGTACAAAAGCCAGATCGATTTCAGC
GAACACGAAGTCAAACTGATCTGCAGCAATCCGGCATTTCGCGAAAAAGTCAACATTGCGAGCCCGTCTCTGGTCGAA
ATGATGGACATCTACATGAAGAACCCGAAGCAGCTGAGCGAAAAGAAAAGCAACGGCCTGAACATCAGCCTGCTGAA
ATACCTGATCCGTAGCAAAGAACGTACCACCCCGTTTGGCTTATTTACCGGCGTTGGTACCGGTTGTTTCAGCAAAAGC
GAGAAGTTCCCGATCCTGATGACCAAGACCGAGAAGAAGGTCAACGTCGATTCTGAGTGGCTGTTTGGCCTGGTTAAC
ATCGTCGAGAAGAACTACGCGGAGAAACTGGAGTTCAAGTTCAACGACGCCTGCTACATCAAAGGCAATCGCGTCATC
CTGGTCTATAGCACCGAAAAAGACGCGGAAGAGATCAGCATCCGTTTCACCAAGGTCTTCAAGGTCCTGTTCGACAAG
ATCAAGGACTACGAGCGCTACGAGAAGTTCATCGAGATCCTGAGCAGCGAATACCCGTCCACCAGCATGAAAAAGATC
AAGCTGTACATCAACGAGCTGATCTCCAAGGGCTTCCTGATTAGCAACCTGCGTCCGAGTTTTAGCAACGCTGACCCGC
TGATGTACTTCATCAAGCAATGCGAGCGCATGGAGATTACCGATATCTGCGAGAAGGCGAACGAGATCTACAAGATGT
GCGAGGATTACAGCAAAACCGACATTGGCAACGGCATCATCAAGTACAACGCGATCAAGACCAAGATGCAGACCCTGT
ATAAGTGCAGCAGCTACCTGCAGGTAGATACCGTTATTGGCGGCGGCGATTTTCAACTGAACCAGGATATCAGCAAAG
CGATTTGCGAAGTTGCGTCTCTGTTCGTCTACCTGAGCAACAGCCCGAAAAAGCAGCACGGTTATCTGGAACATTACCG

CAACAAGTTCATCGAGAAGTACGGCATCGATCGCGAAGTTCCGCTGCTGGAAATGATCGATAGCAGTAACGGTATTGG
CGCACCGACCCCGTATTTAAAACCGCAGAACGACTTCTACGACGAGTACAACACCAAGGACAACTACAAGTACGAGCT
GAAGAACTACTTCCTGGTCGAGTACGAGAAAGCGCTGGCGAATAACAGCTACATCGACATCAACATGGAGACCCTGCA
GAAGATCACCGATTGCACCGTCCAGGAAGAAGAAATTCCGATCAGCCTGGAGCTGTACTTCATCCTGAAGGTCGAAGA
CGGCAAAGTTAGCCTGAATCTGAGTCCGAATTGCGGTAGCTTTGTTGCTGGTAAAACCTTCGGCCGTTTTAGCGTTCAG
AGCGATAACTTCGCGAACGTCCTGAAGAAGTGCAACAAAGAAGAACGCAAAATCCGTAGCCCGCACAGCGAAATCTG
CGAAATCAGCTTCCTGCCGAGTCCGACCCGTAACGGTAATATTGTTCGCACCCTGAGCTTTCGCGAAAAAGAAACCGCG
GTTTTTACCTGCGGCAGCAAAGATAAGAAGGACATCGTCAGCCTGAACGACATCTACATCGGCATCTTCAACGAGAAG
TTCTACGCGCGCGATAAGAAAACCGGCAAGCTGATCATCTTCGAGTCCAACAACATGTACAACCCGATGCTGAACCCG
AACGTCTTTCGCTTTCTGCAGGATATCAGCTACGAAGGTAAACGCGAGTGGTCCGAATTTCCGTGGAGCTACATCTACG
CAGATCTGCGCCATATTCCGACCATCAAGTACAAAGGCATCGTCCTGCAAAACGAGAAGTGGAAGGTCAACATCCAGG
AACTGGAACTGCTGAAGAAGGACTTCGAGTCGTTCAAAGAAAAATTCCTGGCGCTGATTATTGGCCGTAACATGCCGCT
GAACATCTACATTGTTGACGCGGATAACCGCATTCGTCTGAATCTGGCTACCGATCTGAGCATGCGTATTGTCTACGAC
GAGTTCAAGAAGCACAAGGACAGCGATCTGGTCTTCGAGAAAGTCGAAAACGGCAGCGACATCATCTACGATGACGGC
AAAACCTACGCAACCGAAATCGTTGTCCCGCTGTTCCGCAAAAACAAAGAAGAACTGAGCCTGATTCCGCTGAGTCAA
AAGACCTACACCCGCGAACAGCACATGATTCTGCCGTTCAACAACTGGCTGTACCTGAAGCTGTACTGCAACGAAAAC
CGCGAAGAAGAGCTGATCGCCTTCTACATCATGGACTTCTACGAGAGCCTGAAAGAGAAATACGGCATCTCCTACTTCT
ACATGCGCTACGCGGATCCGAAACCGCATATTCGTCTGCGTTTACACGCAACCCGCGAATTACTGCTGCAGGTTTATCC
GCAAATCCTGAAGTGGTACAGCGAACTGTTCAGCGATCAGATTGTCGGCGATATGACCATCAGCGTCTACGATCGCGA
AATTGAACGTTACGGCGGCGCTTTACTGATGGATACCGCGGAAAAAGTCTTCTTCGAGGACAGCTACATCGTCGAAAA
CATCCTGCGTCTGAAACGTCTGGGCAAAATTAGCCTGAACCTGGACGACGTTGCAGTTGTCTCCATCATCATGTACGTC
AGCCAGTTCTACAACAAGTACGAAGAACAGCTGCAGTTCCTGAGCATTAACTACCACAGCAGCGACTTCATCAGCGAG
TTCAAGAAGAAGAAGGACAACCTGCTGCGCATCTGCGATATCGAAAACGAGTGGAACAACCTGAACAGCATGAAGGA
CGGCAAGGTTGTCTACGAACTGATGTGGCGTCGTTGCAAAGTCATCAGCGAGTACAGCGAGAAGATCCGCAAAATCAA
CCCGGATCCGATGTTCAAGAACAGCATTGTCGCGAGCGTCATCCATCTGCATTGCAACCGTCTGATCGGTACCAATCGC
GAACTGGAACGCAAACTGATGGCATTCGCGGAAAGCGTTCTGTACGCGAAAAAGTACGTCATGCGCCGTATCGAAGTT
AACGGCAAAAAGTAA

>BpcC, codon optimized

ATGGACAACAGCATCATCAGCATCGTCAAAGAGATTGCGCGTAACCTGGGCGATTACGATAACGTCAAACGCATCGTC
GCGGACAAAGATAACATCCGCGTCATGGGCGAGTTCAACTTTCAAGGTTGGGAACCGCTGACCCTGAGTCACGGTATT
CCGGGTATTTGTCTGCTGTACGGCAAACTGATGGAGTGCTTTCCGGACGAAGAAATCTGGGCAGAACTGGCACATCAGT
ATCTGGGGTATCTGGTCCAGGAGATCAACAAAGAGGGCTTTCAAACCCTGAGCATGTTTAGCGGTACCTCTGGTATTGG
TCTGGCAGTTGCATCAGTCAGCAACAACTTCTGCAACTACAACAAACTGCTGAACACCATCAACAGCTACATCATCAAC

TGGTTCGACGAGTTCATCGACAGCATCGATCTGAAAAAAGGCACCCGTAGCATCTGCTACGACGTTATTGAAGGCCTGA
GCGGCATTCTGAGCTATTGCAGCATCTACTACGAGCAGGAAAGCTTTAGCCCGATTCTGCTGAAAGGCCTGAAAAAACT
GGTCGAGCTGACCTACGACATCGAAGTCAAAGGCTATCACGTTCCGGGTTGGTATATTCCGAGCGACAACCAGTTCAGC
ACCGTTGAAAAAGAGCTGTACCCGTACGGTAACTTCAACACCAGCTTCAGCCACGGCATTGCAGGTCCGCTGACCCTGC
TGAGCGAAATGAAATCCAAAGGCTTCATGATCGAGGGCCAGGAAGAAGCGATCGAGAAAATCGTCAAATTCCTGTTCG
ACTTCCGCAGCAACGATCAGAAACGCGACTTTTGGAAAGGCCAGATCGATTTCCACGAATACATCACCGGCAAAGTCA
GCGAGAAAAACATCATCCGTCGCGACGCTTGGTGTTACGGTAATCCGGGCGTTTGCTATTCTCTGATCATGGCTGGCAA
CGCGATGAAAAACCAGAGCTGGATCGACTACGGCATCCACAACATGAAAAAGACCCTGAGCGACGTCAAAGGCATCTT
TAGCCCGACCTTTTGCCACGGTTTTTCCGGTCTGTACCAGGTTGTCAACAGCATCGAATTCACCATCGGCAAAGACATCT
TCTACAGCGAGAAAAAAGAGCTGCTGAACAAAATCATGAGCTTCTACGACAGCAACTACATCTTCGGCTTCCGCAACA
TGGAAGTAGGCGACGAAAACGGCAACATTCGCGCATTTGAACATCTGGGTCTGCTGGACGGTACCATTGGCGTTTGTCT
GGCACTGCTGGAAGGCGAACACAAAACCAAAAACATCTGGAAACGCGCATTTCTGCTGGCATAA

References

1. Walker, M. C.; Eslami, S. M.; Hetrick, K. J.; Ackenhusen, S. E.; Mitchell, D. A.; van der Donk, W. A., Precursor peptide-targeted mining of more than one hundred thousand genomes expands the lanthipeptide natural product family. *BMC Genomics* **2020**, *21* (1), 387.
2. Tietz, J. I.; Schwalen, C. J.; Patel, P. S.; Maxson, T.; Blair, P. M.; Tai, H. C.; Zakai, U. I.; Mitchell, D. A., A new genome-mining tool redefines the lasso peptide biosynthetic landscape. *Nat. Chem. Biol.* **2017**, *13* (5), 470-478.
3. Lowe, T. M.; Eddy, S. R., tRNAscan-SE: a program for improved detection of transfer RNA genes in genomic sequence. *Nucleic Acids Res.* **1997**, *25* (5), 955-64.
4. Chan, P. P.; Lowe, T. M., tRNAscan-SE: Searching for tRNA Genes in Genomic Sequences. *Methods Mol. Biol.* **2019**, *1962*, 1-14.
5. Sorbara, M. T.; Littmann, E. R.; Fontana, E.; Moody, T. U.; Kohout, C. E.; Gjonbalaj, M.; Eaton, V.; Seok, R.; Leiner, I. M.; Pamer, E. G., Functional and genomic variation between human-derived isolates of *Lachnospiraceae* reveals inter- and intra-species diversity. *Cell Host Microbe.* **2020**, *28* (1), 134-146.e4.
6. Wu, C.; Lower, B. A.; Moreira, R.; Dorantes, D.; Le, T.; Giurgiu, C.; Shi, Y.; van der Donk, W. A., Investigation into the mechanism of action of the antimicrobial peptide epilancin 15X. *Front. Microbiol.* **2023**, *14*.
7. Hein, R.; Uzundal, C. B.; Hennig, A., Simple and rapid quantification of phospholipids for supramolecular membrane transport assays. *Org. Biomol. Chem.* **2016**, *14* (7), 2182-5.
8. Seemann, T., Prokka: rapid prokaryotic genome annotation. *Bioinformatics* **2014**, *30* (14), 2068-9.
9. Eddy, S. R., Accelerated Profile HMM Searches. *PLoS Comput Biol* **2011**, *7* (10), e1002195.
10. Shi, Y.; Yang, X.; Garg, N.; van der Donk, W. A., Production of lantipeptides in *Escherichia coli*. *J. Am. Chem. Soc.* **2011**, *133* (8), 2338-41.
11. Brademan, D. R.; Riley, N. M.; Kwiecien, N. W.; Coon, J. J., Interactive peptide spectral annotator: A versatile web-based tool for proteomic applications. *Mol. Cell. Proteom.* **2019**, *18* (8, Supplement 1), S193-S201.
